# Unveiling the charge transfer dynamics steered by built-in electric fields in BiOBr photocatalysts

**DOI:** 10.1038/s41467-022-29825-0

**Published:** 2022-04-25

**Authors:** Zhishan Luo, Xiaoyuan Ye, Shijia Zhang, Sikang Xue, Can Yang, Yidong Hou, Wandong Xing, Rong Yu, Jie Sun, Zhiyang Yu, Xinchen Wang

**Affiliations:** 1 State Key Laboratory of Photocatalysis on Energy and Environment, College of Chemistry, Fuzhou University, Fuzhou, Fujian 350108 China; 2 College of Chemical Engineering, Fuzhou University, Fuzhou, Fujian 350108 China; 3 National Center for Electron Microscopy in Beijing, School of Materials Science and Engineering, Key Laboratory of Advanced Materials of Ministry of Education of China, State Key Laboratory of New Ceramics and Fine Processing, Tsinghua University, Beijing, 100084 China; 4 Fujian Science & Technology Innovation Laboratory for Optoelectronic Information of China, Fuzhou 350100, China and College of Physics and Information Engineering, Fuzhou University, Fuzhou, 350100 China

**Keywords:** Photocatalysis, Photocatalysis, Nanoscale materials

## Abstract

Construction of internal electric fields (IEFs) is crucial to realize efficient charge separation for charge-induced redox reactions, such as water splitting and CO_2_ reduction. However, a quantitative understanding of the charge transfer dynamics modulated by IEFs remains elusive. Here, electron microscopy study unveils that the non-equilibrium photo-excited electrons are collectively steered by two contiguous IEFs within binary (001)/(200) facet junctions of BiOBr platelets, and they exhibit characteristic Gaussian distribution profiles on reduction facets by using metal co-catalysts as probes. An analytical model justifies the Gaussian curve and allows us to measure the diffusion length and drift distance of electrons. The charge separation efficiency, as well as photocatalytic performances, are maximized when the platelet size is about twice the drift distance, either by tailoring particle dimensions or tuning IEF-dependent drift distances. The work offers great flexibility for precisely constructing high-performance particulate photocatalysts by understanding charge transfer dynamics.

## Abstract (web summary)

While internal electric fields alter charge-separation dynamics in solar-to-chemical conversions, a greater understanding of such processes is necessary. Here, authors analyze charge transfer dynamics modulated by built-in electric fields and identify carrier drift distances as a critical parameter.

## Introduction

Solar-driven photocatalytic reactions offer great promises for environmental remediation and renewable energy generation by converting inexhaustible solar energy into clean and environmentally benign fuels^1–3^. Generally, three major steps are involved in photocatalytic reactions: (1) excitation of electron-hole pairs within a particulate semiconductor; (2) migration of charge carriers to the catalyst surface; and (3) surface redox reactions^4^. Photocatalytic systems often suffer from poor solar energy conversion efficiency because of the intrinsically strong propensity of charge carrier recombination in the second step^5^. Therefore, it is imperative to develop strategies to manipulate carrier migration with great controllability to inhibit carrier recombination. The construction of internal electric field (IEF) by surface tailoring and interface engineering approaches, including co-catalyst loading^6,7^, phase junctions^8–14^, heterojunctions^15–18^, and facet junctions^19–22^, could precisely manipulate the migration of photo-generated electrons (e^-^) and holes (h^+^) to spatially separated reductive and oxidative sites, thereby enhancing the photocatalytic performance^23–29^.

Knowledge of the charge migration properties, including the diffusion length and the IEF-dictated drift distance of non-equilibrium charge carriers, guides the rational design of particulate photocatalysts^30^. Charge transport behavior in semiconductors is typically characterized by transient absorption^31^, photoluminescence-quenching measurements^32^, and electrical characterization^33^, where the diffusion constants and lifetimes of carriers could be determined^34–37^. However, these measurements were only applicable to thin-film electrodes with external electric fields, which differs from the carrier diffusion dynamics modulated by IEF produced at the semiconductor-liquid interface for power photocatalysts in the solvent^38,39^. A comprehensive understanding of the collective migration of photo-excited non-equilibrium carriers steered by IEFs at the semiconductor/solution or semiconductor/cocatalyst interfaces still remains unexplored^34,40–43^.

Bismuth oxyhalide (BiOX, X = Cl, Br, I) photocatalysts have attracted widespread attention due to their unique layered structure consisting of fluorite-like [Bi_2_O_2_] units sandwiched by two [X]^−^ slabs^44–48^. The strong intralayer covalent bonding along with the 2D layer and the weak interlayer van der Waals interaction perpendicular to the 2D layer gives rise to a lamellar growth habit^46^, which endows them with anisotropic carrier transfer properties^49^. It was reported that the anisotropic facets can drive photo-generated electrons and holes to {001} reductive and {110} oxidative sites^44–46,50^, forming a {001}/{110} binary facet junction in the BiOBr platelets^30^. Herein, we take advantage of the well-defined morphology of BiOBr platelets and the cascade binary junctions to track the anisotropic carrier transport dynamics steered by IEFs, using metal and oxide nanoparticles as reduction and oxidation probes^20^. Two contiguous IEFs at the (001) facet/solution and (200) facet/solution junctions could efficiently guide electrons and holes to (001) and (200) surfaces, respectively. The spatial distribution of photo-deposited metal nanoparticles is found to exhibit a characteristic Gaussian curve. An analytical model, modified from the Haynes-Shockley model^51–53^, satisfactorily explains the Gaussian curve and allows us to identify two important parameters, including the drift distance of electrons influenced by IEFs and the diffusion length of electrons, which is useful for the rational design of BiOBr platelets with promoted photocatalytic efficiency. The overall photocatalytic performance is optimized either by tailoring particle sizes or regulating IEF-relevant drift distances of electrons, when the platelet size is about twice the drift distance. Our study demonstrates the capacity of a full utilization of solar energy by precisely guiding a unidirectional and steady charge flow. Our findings propose a conceptual strategy to measure charge carrier transport modulated by IEFs, which could be extended to a broad spectrum of particulate photocatalytic systems, especially in nanocomposites containing heterogeneous interfaces with IEFs, deepening insights into the intrinsic transport properties of charge carriers in semiconductor photocatalysts.

## Results

### Synthesis of well-defined BiOBr platelets enclosed by anisotropic facets

**Fig. 1:**
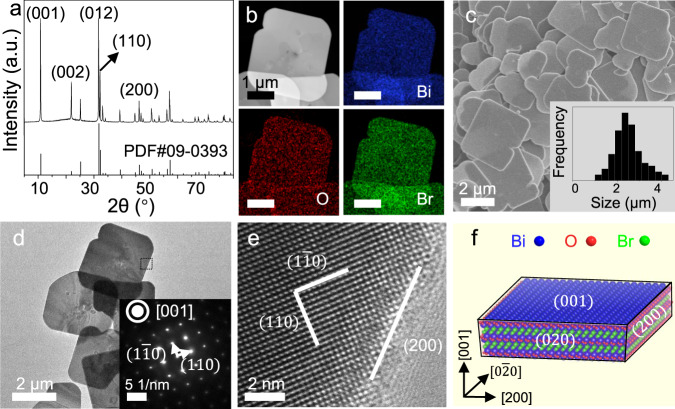
Microscopic characterization of BiOBr platelets. **a** XRD pattern, **b** EDS maps, **c** SEM morphology (inset: statistical histogram of the lateral size distributions of BiOBr platelets), **d** TEM image of BiOBr platelets. A typical selected-area electron diffraction (SAED) pattern of BiOBr platelets is given in the inset. **e** Enlarged HRTEM image recorded at the edge of a platelet. **f** Schematic diagram depicting the crystal habit of BiOBr platelets. Source data are provided as a Source Data file.

BiOBr photocatalysts were synthesized by a hydrothermal method (see Method section for details). X-ray diffraction (XRD) pattern (Fig. 1a) of platelets is ascribed to a tetragonal phase of BiOBr (PDF #09–0393). Energy-dispersive X-ray spectroscopy (EDS) maps reveal the homogenous distribution of Bi, O, and Br elements within platelets (Fig. 1b). Scanning electron microscopy (SEM) and transmission electron microscopy (TEM) images show that the BiOBr platelets exhibit well-defined square morphology, with a mean lateral size and thickness of 2.5 μm and 110 nm, respectively (Fig. 1c, d and Supplementary Fig. 1). Selected-area electron diffraction (SAED) (inset of Fig. 1d) confirms that the platelets are single crystalline. Since the electron beam direction is perpendicular to their basal planes, the basal facets are ascribed to be (001) planes. High-resolution electron microscopy (HREM) imaging was performed at the platelet edge (Fig. 1e), demonstrating that the side faces are terminated by (200) and (020) planes. These results combined indicate that BiOBr crystals are enclosed by two large {001} facets as the basal planes and four identical {200} planes being the side faces, as illustrated in Fig. 1f.

### Intrinsically facet-dependent carrier separation behavior

**Fig. 2:**
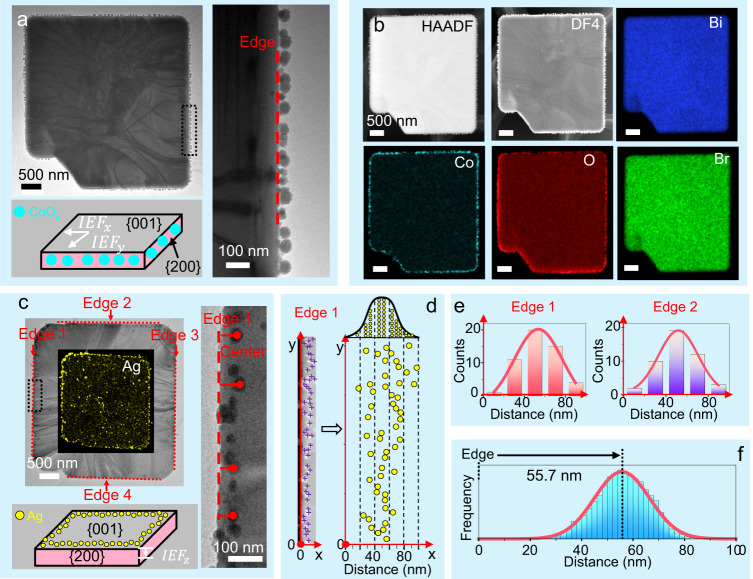
Anisotropic charge separation behavior. **a** Low magnified, expanded TEM images and schematic illustration indicating CoO_x_ nanoparticles are dominantly photo-deposited on the side faces of a BiOBr platelet, as evidenced by the EDS maps in **b**, **c** Overview image and enlarged TEM image showing the photo-deposition of Ag nanoparticles on the (001) basal facets, with a schematic diagram given in the bottom left panel (inset: EDS map). **d** Left panel: HAADF image recorded at Edge 1 of **c** showing the aggregation of Ag nanoparticles in a line pattern. The centers of Ag nanoparticles are marked by purple crosses. Right panel: Their positions are plotted in a coordinate system. **e** Statistical histograms of the distances from the centers of Ag nanoparticles to edge 1 or edge 2, which can be described by a Gaussian profile. **f** Statistical analysis of the spatial distribution of Ag nanoparticles with respect to the platelet edges after counting more than 50 individual BiOBr platelets from multiple TEM images. Source data are provided as a Source Data file.

To investigate the carrier separation nature, photo-deposition of CoO_x_ (as hole probes) and Ag (as electron probes) co-catalysts were employed to identify the oxidation and reduction reactive facets, respectively^20,49^. The enlarged TEM image in Fig. 2a shows that a multitude of nanobeads is photo-deposited on {200} side faces of BiOBr platelets. Those nanobeads show bright high-angle annular dark-field (HAADF) contrast and are ascribed to CoO_x_ co-catalysts, as evidenced by Co and O EDS maps in Fig. 2b. TEM and EDS observations from a side view (Supplementary Fig. 2) further confirm the preferential deposition of CoO_x_ nanoparticles on the side faces, indicating that the photo-generated holes are spontaneously driven to the photo-oxidative {200} facets by lateral IEFs (defined as ${{IEF}}_{x}$ and ${{IEF}}_{y}$) in the space charge regions, as schematically depicted in Fig. 2a. The direction of lateral IEFs is pointing against the {200} facets.

In contrast, photo-excited electrons have a propensity to accumulate around the platelet edges (the four side faces of the BiOBr platelet are defined as edge 1–4, see the dashed line in Fig. 2c), forming distinct line patterns that are tens of nanometers away from the edge, as clearly shown in the enlarged TEM image (the right panel of Fig. 2c). This indicates that the photo-generated electrons are predominantly driven to the (001) surfaces by a vertical IEF (defined as ${{IEF}}_{z}$, see schematic illustration in the bottom left panel of Fig. 2c).

To quantify the spatial distribution characteristics of Ag particles, the centers of Ag nanoparticles near edge 1 were extracted from the HAADF image in the left panel of Fig. 2d and their positions were superimposed on a coordinate system (the right panel of Fig. 2d). When the positions of Ag nanoparticles are projected along the X-axis, an analysis of the statistical histogram reveals an interesting Gaussian distribution, which holds for the Ag particles near all the edges (edge 1 and edge 2 as two typical examples, Fig. 2e). In the end, the spatial distribution of Ag particles was summarized from more than 50 individual BiOBr platelets, exhibiting a prominent Gaussian curve centering around 55.7 nm (Fig. 2f).

For comparison, CoO_x_ and Ag co-catalysts were also loaded onto BiOBr platelets via a wet impregnation method. Both CoO_x_ and Ag nanoparticles are homogeneously deposited on both {200} side faces and {001} basal planes (Supplementary Figs. 3 and 4), indicating the physical adsorption of co-catalysts does not exhibit facet selectivity.

### Carrier diffusion pathway modulated by lateral IEFs

**Fig. 3:**
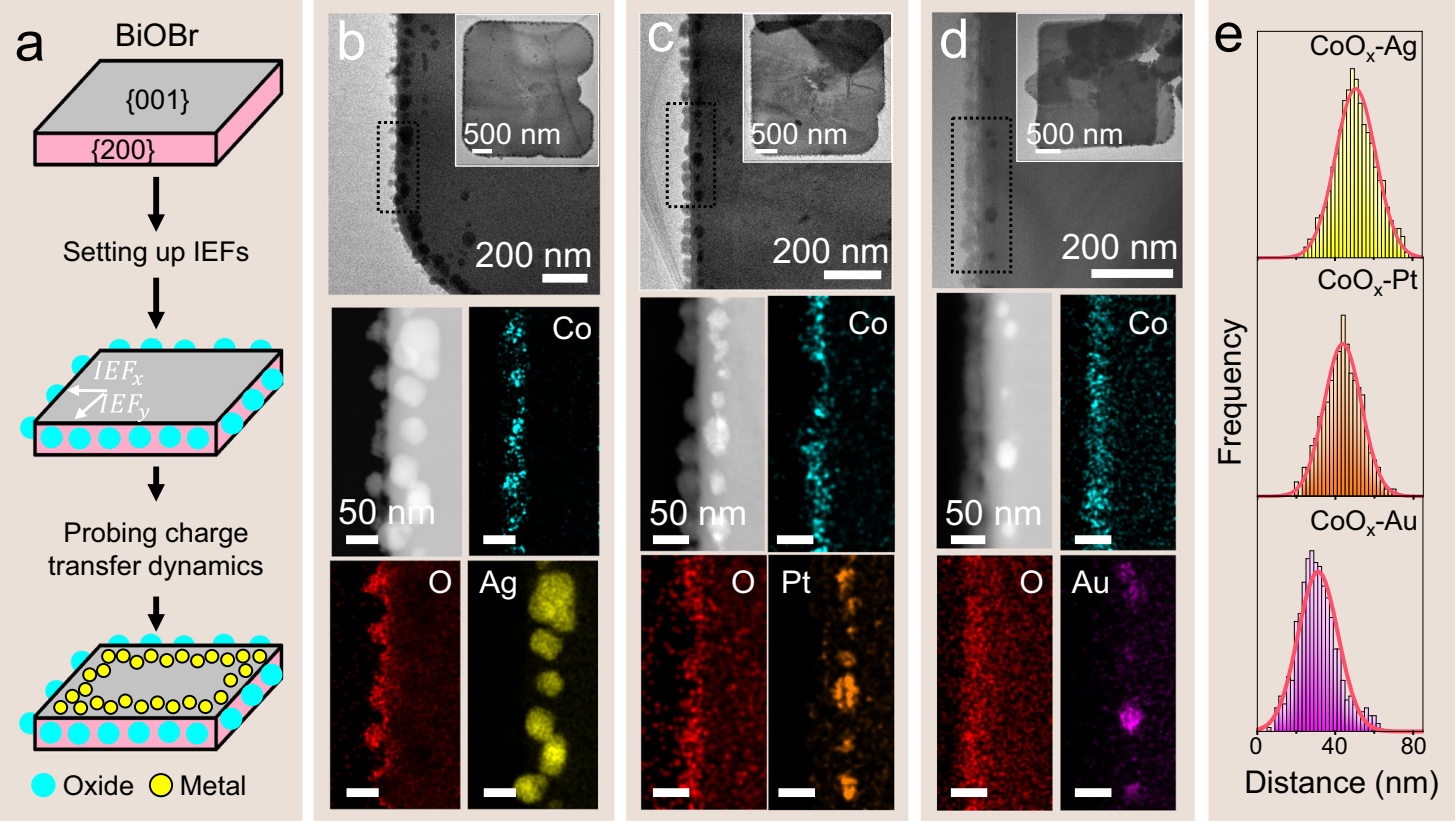
The collective charge transfer pathway steered by IEFs. **a** Schematic illustration showing the construction of IEFs by photo-deposition of CoO_x_ co-catalysts on the surface of BiOBr platelets, where a variety of metal species were employed as the probe to track the transfer pathway of electrons. TEM images and EDS maps show the photo-deposition of dual co-catalysts in the order of CoO_x_ co-catalysts and Ag (**b**), Pt (**c**), or Au (**d**) nanoparticles. These samples are termed as CoO_x_-metal (Ag, Pt, or Au)/BiOBr. **e** Statistical histograms of the distances from the centers of metal nanoparticles to the BiOBr platelet edges, including the CoO_x_-Ag, CoO_x_-Pt, and CoO_x_-Au pairs. Source data are provided as a Source Data file.

The IEF exerts an electric force to the carriers, which influences the migration and modulates their spatial distribution in the same direction. The unique platelet geometry enables us to probe the effect of lateral IEFs on the lateral migration dynamics of electrons by tracking the spatial distribution of metal particles on {001} facets. A widely employed approach, *i.e*., the photo-deposition of metal oxide co-catalysts (CoO_x_ and MnO_x_) on the {200} side faces, was applied to simulate IEFs with varied strengths, and photo-deposited Ag, Pt, and Au nanoparticles were used as probes to track the dynamic migration of photo-excited electrons steered by lateral IEFs (Fig. 3a). Those samples are named after the photo-deposition sequence, such as CoO_x_-Ag/BiOBr, CoO_x_-Pt/BiOBr, CoO_x_-Au/BiOBr pairs.

Extensive TEM imaging and EDS mapping were performed. Metal nanoparticles (Ag, Pt, and Au) line up on the (001) facets (Fig. 3b–d). Statistical analysis (Fig. 3e and Table 1) indicates that the spatial distribution of all the experimental batches exhibits a characteristic feature in terms of a Gaussian profile. This also holds for the MnO_x_-Ag, Pt, and Au system (Supplementary Fig. 5 and Supplementary Table 1).

**Table 1:** A summary of the lateral drift distance, diffusion length, and average thickness for several metal nanoparticles/BiOBr photocatalysts groups.

Batches	Lateral drift distance (${L}_{{{IEF}}_{x}}$, nm)	Standard deviation ($\sigma$, nm)	Diffusion length (${L}_{e}$, nm)	Mean thickness (nm)
Ag/BiOBr-2.5	55.7	10.7	7.6	110
Ag/BiOBr-0.5	48.7	10.3	7.3	48
Ag/BiOBr-0.1	51.8	10.5	7.4	40
CoO_x_-Ag/BiOBr-2.5	50.7	10.4	7.4	110
CoO_x_-Pt/BiOBr-2.5	43.7	9.6	6.8	110
CoO_x_-Au/BiOBr-2.5	31.2	10.3	7.3	110

Source data are provided as a Source Data file.

In principle, a change in pH value affects the redox potential of the solution, which in turn modulates the IEF strength at the solution/semiconductor interface. Accordingly, a series of parallel experiments were designed to elucidate the influence of IEF strength on the spatial distribution of Pt nanoparticles by varying the pH values of the solution, such as Pt(pH = 7)/BiOBr, Pt(pH = 4)/BiOBr, and Pt(pH = 1)/BiOBr pairs. TEM, HAADF images, EDS maps, and statistical analysis (Supplementary Fig. 6a–c) reveal that the photo-deposited Pt nanoparticles still follow the similar Gaussian distribution profile (Supplementary Fig. 6d and Supplementary Table 1). More importantly, the positions of Gaussian centers shift to the right as pH decreases, establishing a direct correlation between the drift distance of the charge carrier and the IEF strength. Hence, it is the IEF at the semiconductor/solution interface that steers the collective migration of charge carriers via a general mechanism.

### Theoretical modeling of the dynamic migration of photo-generated carrier modulated by IEFs

**Fig. 4:**
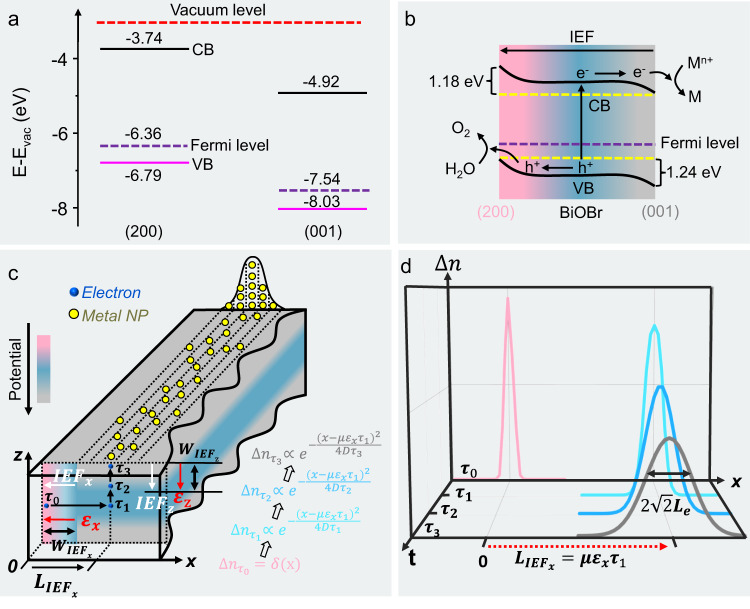
The mechanism of charge separation and transfer. **a** Schematic diagram of electronic band structures for (200) and (001) facets by DFT calculations. CB: conduction band, VB: valence band. **b** Schematic illustration of the migration pathway of photogenerated electrons and holes within the (200)/(001) facet junction. **c** Schematic illustration of the cross-section of a platelet showing the migration pathway of photo-generated electron driven by two IEFs (horizontal and vertical). The directions of the intrinsic electric fields are denoted by red arrows. **d** The spatial distribution profile of electrons at different time sequences.

Theoretical analysis was conducted to understand the origin of IEFs and their influence on the dynamic migration of carriers. As confirmed by the density functional theory (DFT) calculations (the computational methods are described in the Method section)^54–58^. The conduction band (CB) and valence band (VB) edge follow the order of {200}> {001} (Fig. 4a and Supplementary Fig. 7). Thus, a {200}/{001} binary facet junction (defined as a homojunction between neighboring facets in a single-crystalline semiconductor, Supplementary Fig. 8)^8–10^ can be formed in BiOBr platelets, leading to well-matched cascade band structures between the two facets and the bulk (Fig. 4b). This produces two IEFs (horizontal versus vertical), a lateral one pointing left (${{IEF}}_{x}$), and a vertical one pointing upward (${{IEF}}_{z}$, Fig. 4c), which provides the driving forces for the experimentally observed facet-dependent carrier separation behavior.

Now we start to model the effect of IEFs imposed either by metal oxide nanoparticles or solution to carrier migration, with a schematic carrier transfer pathway of BiOBr platelet given in cross-section view (Fig. 4c). The migrations of electrons within three zones, (1) space charge region on the (200) surface with lateral ${{IEF}}_{x}$, (2) in the bulk, (3) space charge region on the (001) facet with vertical ${{IEF}}_{z}$, are taken into consideration. In the first zone, electrons and holes are produced in a form of a pulsed signal at ${{{{{{\rm{\tau }}}}}}}_{0}$ ($\triangle n=\delta ({{{{{\rm{x}}}}}})$ where $\triangle n$ is the spatial distribution function of electrons and $\delta \left({{{{{\rm{x}}}}}}\right)$ is a Dirac delta function). Then, electrons are directed into the bulk by a force imposed from the lateral ${{IEF}}_{x}$ with an interaction time of ${{{{{{\rm{\tau }}}}}}}_{1}$. Based on the well-established Haynes-Shockley experiment (Supplementary Fig. 9a)^51–53^ that depicts the interaction of carrier with an external electric field, the modulation can be described using Eq. (1), where $\mu$, ${\varepsilon }_{x}$ and $D$ corresponds to the carrier mobility, the strength of IEF, and diffusion constant, respectively.

1 $$\triangle {n}_{1}\propto {e}^{-\frac{{(x-\mu {\varepsilon }_{x}{\tau }_{1})}^{2}}{4D{\tau }_{1}}}$$

This corresponds to a Gaussian curve (Supplementary Fig. 9b). Then, the electrons drift in the bulk without the influence of ${{IEF}}_{x}$ at ${{{{{{\rm{\tau }}}}}}}_{2}$. Finally, they are spontaneously guided to the (001) facets by the vertical ${{IEF}}_{z}$ at ${{{{{{\rm{\tau }}}}}}}_{3}$. For the last two stages, the lateral spatial distribution of electrons is not shifted due to the absence of lateral ${{IEF}}_{x}$. The electron profile is equivalent to a convolution of Eq. (1) with a Gaussian function after given a drifting time (Supplementary Fig. 9c), as shown in Eq. (2).

2 $$\triangle {n}_{3}\propto {e}^{-\frac{{(x-\mu {\varepsilon }_{x}{\tau }_{1})}^{2}}{4D{\tau }_{3}}}$$

Equation (2) exhibits a Gaussian profile distribution (Fig. 4d), where the Gaussian peak center, $\mu {\varepsilon }_{x}{\tau }_{1}$, could be treated as the drift distance (defined as ${L}_{{{IEF}}_{x}}$) driven by the lateral ${{IEF}}_{x}$ in the space charge region (defined as ${W}_{{{IEF}}_{x}}$ and ${{W}_{{IEF}}}_{y}$ in the following context) and the standard deviation ($\sigma$) is $\sqrt{2}$ times of the diffusion length of electrons ($\sqrt{D{\tau }_{3}}$ is defined as ${L}_{e}$), because ${{{{{{\rm{\tau }}}}}}}_{3}$ is the overall lifetime of electrons.

The actual carrier distribution profile is more complex, as electrons might be excited from any site in the space charge region near {200} facets and form a Gaussian curve distribution. The overall distribution can be considered as a convolution of a Gaussian function with a plateau function (Supplementary Fig. 10). Two extreme cases are considered here. For a simple case, when the width of a lateral space region (${W}_{{{IEF}}_{x}}$) is negligible compared to the diffusion length of electrons, the carrier retains a Gaussian profile. The mathematical fitting of the Gaussian curve gives ${L}_{{{IEF}}_{x}}$ and ${L}_{e}$ (Supplementary Fig. 10b). Under the circumstance that ${W}_{{{IEF}}_{x}}$ is comparable or even larger than the ${L}_{e}$, the carrier distribution starts to exhibit a plateau feature in the center while the Gaussian damping feature is preserved at the edge of the plateau (Supplementary Fig. 10c). The center of the peak corresponds to ${L}_{{{IEF}}_{x}}$. However, more sophisticated mathematics analysis needs to be treated to deconvolute ${L}_{{{IEF}}_{x}}$ and ${L}_{e}$. For the carriers produced in the second and third zones, electrons are evenly excited and drift to the (001) facets, leading to a homogeneous distribution profile and contributing to a uniform background to the carrier distribution profile. Hence, it is the modulation of two contiguous IEFs (horizontal versus vertical) that results in a collective migration of carriers exhibiting a distinct Gaussian profile.

As presented in Figs. 2 and 3, for our cases, statistical analysis reveals that the spatial distribution of metal particles has a Gaussian curve without evident plateau, implying that the width of a lateral space charge layer (${W}_{{{IEF}}_{x}}$) is small. Hitherto, the lateral drift distance imposed by lateral ${L}_{{{IEF}}_{x}}$ and ${L}_{e}$ is determined in Table 1 and Supplementary Table 1 by fitting Gaussian functions to the spatial distribution of metal nanoparticles (Figs. 2, 3 and Supplementary Figs. 5, 6), which offers important insights into the carrier migration dynamics in BiOBr platelets. First, by using an electron energy loss spectroscopy (EELS) technique^59–61^, we revealed a weak correlation between charge dynamic and thickness for identical BiOBr-2.5 platelets, implying that a change in thickness of BiOBr platelets has little effect on the charge dynamic (Supplementary Figs. 11–13). Secondly, ${L}_{{{IEF}}_{x}}$ varies as the origin of IEFs changes, consistent with the fact that different solutions or co-catalysts impose varied IEFs in strength. Third, ${L}_{{{IEF}}_{x}}$ existing at the semiconductor/solution interfaces or semiconductor/metal oxides (CoO_x_ or MnO_x_) is nearly on the same scale, ranging from 30 to 55 nanometers. It indicates that those IEFs have close electric strength. Fourth, the intrinsic diffusion length for electrons (${L}_{e}$) along the [100] direction is estimated at ca. 7 nm, which is independent of the origin and the strength of IEFs. Fifth, by comparing the CoO_x_-Ag, Pt, Au, and MnO_x_-Ag, Pt, Au pairs, one might expect that Ag, Pt, and Au nanoparticles, as probes of photo-generated electrons, might have the same distribution, because the lateral ${{IEF}}_{x}$ provided by CoO_x_ or MnO_x_ particles does not change. Unexpectedly, the Gaussian center of Pt nanoparticles is about 10 nm closer to the platelet edge than that of Ag nanoparticles. It implies that a change in the status of the solution may affect the space charge regions on both (001) and (200) facets and modify the migration dynamics of electrons. Last but not least, decreasing pH in the solution environment could effectively increase the drift distance. For instance, the drift distance of Pt (pH = 1)/BiOBr is ~19 nm farther away compared to Pt (pH = 7)/BiOBr batch, using the platelet ledge as a reference. It demonstrates that IEFs do play a vital role in dictating the spatial distribution of carriers.

The current methodology described here is extensible to similar systems, e.g., BiOCl platelets (Supplementary Fig. 14) to determine the intrinsic carrier transport properties, such as carrier diffusion length and drift distance imposed by IEFs. Those parameters serve as important guidelines to enhance the quantum efficiency by rational structure optimization, e.g., regulating the size^62^ and morphology of photocatalysts^5,63^.

### Structure optimization of BiOBr platelets with enhanced photocatalytic performance

Structure optimization was performed by tailoring the sizes of BiOBr platelets. Four batches of BiOBr platelets, with a mean lateral size of 2500, 500, 100, and 50 nm denoted as BiOBr-2.5 (Fig. 1c), BiOBr-0.5, BiOBr-0.1, and BiOBr-0.05 (Supplementary Fig. 15) were fabricated. The optical absorption property and different synthetical methods of BiOBr platelets have a minor effect on their charge dynamics and photocatalytic performance (Supplementary Figs. 16–18). Thus, we focused on investigating the effect of lateral sizes on their charge dynamic behavior. Ag nanoparticles could also be photo-deposited on the (001) surface of BiOBr-0.1 and BiOBr-0.5 platelets and their drift distance/diffusion length were measured as 51.8/7.4 and 48.7/7.3 nm, respectively (Supplementary Fig. 19 and Table 1), which were close to that of BiOBr-2.5 platelets (55.7/7.6 nm). It implies that the charge separation process of photo-deposition is independent of their lateral sizes. However, Ag nanoparticles were not selectively photo-deposited on the surface of BiOBr-0.05 platelets due to a reduction of platelet thickness (mean thickness: 17 nm, Supplementary Fig. 20). This might decrease the width of the space charge region in the thickness direction, weakening the strength of ${{IEF}}_{z}$^64^ and leading to a random distribution of Ag nanoparticles on the (001) surface of BiOBr-0.05 platelets (Supplementary Fig. 20).

**Fig. 5:**
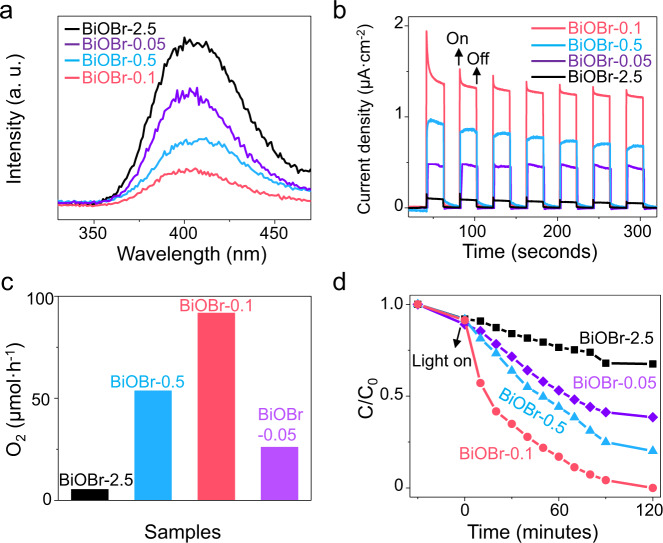
The photocatalytic performance of BiOBr platelets with different lateral sizes. **a** The photoluminescence (PL) spectra, **b** chronoamperometry (CA) curve spectra, **c** the performance of photocatalytic oxygen evolution, and **d** photocatalytic reduction of Cr(VI) to Cr(III) for BiOBr-2.5, BiOBr-0.5, BiOBr-0.1, and BiOBr-0.05 platelets, respectively. Source data are provided as a Source Data file.

Next, a series of photoelectric and photoelectrochemical characterization were employed to investigate their charge separation efficiencies. Photoluminescence (PL) intensity drops sharply as the lateral size decreases at first and the highest charge separation efficiency was achieved when the size was about 100 nm (Fig. 5a). It was worth noting that, when the lateral size was reduced to 50 nm, increased fluorescence intensity was observed, which might be caused by the reduced strength of directional IEFs. In addition, the BiOBr-0.1 photocatalyst existed the longest fluorescence lifetime due to its suitable lateral size (Supplementary Fig. 21). The photocurrent density revealed that the separation capacity of photogenerated carriers follows the order of BiOBr-0.1 > BiOBr-0.5 > BiOBr-0.05 > BiOBr-2.5 (Fig. 5b), and electronic impedance spectroscopy (EIS) characterization measurements demonstrated the same trend (Supplementary Fig. 22). Furthermore, the photocatalytic water oxidation and Cr(VI) reduction reactions were carried out. As shown in Fig. 5c, d, the photocatalytic oxygen evolution rate of BiOBr-0.1 platelets is 92.0 μmol h^−1^, which is 16.4 times higher than that of BiOBr-2.5 platelets and the degradation rate of Cr(VI) also increases when the lateral size of BiOBr platelets is 100 nm. Interestingly, as we manipulate the drift distances by adjusting the IEF strengths at varied pH values, the water oxidation evolution performance of Pt-BiOBr-0.1 platelets is further improved (Supplementary Fig. 23). This offers great promise to optimize the performance of photocatalysts by matching the drift distance with fixed particle size, simply via optimizing the reaction conditions, such as pH values.

## Discussion

As summarized in Table 1 and Supplementary Table 1, the drift distances of carriers steered by IEFs range from about 30 to 55 nm, and the diffusion length of electrons is ca. 7 nm in the BiOBr platelets. The abovementioned characterizations suggest that the charge separation efficiency, as well as photocatalytic performances, can be maximized when the particle size (~100 nm) is close to twice the drift distance of carriers (${L}_{{{IEF}}_{x}}$). This serves as a guideline for the rational design of BiOBr platelets. Notably, it is different from previous knowledge that the size of photocatalysts should be two times the width of the space charge region to maximize light absorption and charge carrier separation for photocatalytic redox reactions^23^.

In the end, we also photo-deposited Ag nanoparticles on the surfaces of BiVO_4_ photocatalysts with {010}/{110} facet junctions, where Ag nanoparticles segregated on the surfaces in line patterns (Supplementary Fig. 24). However, for WO_3_, ZnO, CeO_2_, CdS, and Ta_3_N_5_ photocatalysts without spatially separated reductive and oxidative surfaces, the distribution of Ag nanoparticles was random (Supplementary Fig. 25). Those facts explicitly suggest that the current methodology to quantitatively analyze the collective charge transfer dynamics modulated by built-in electric fields is mainly applicable to photocatalyst containing photo-reduction and photo-oxidation facet junctions^8–10,20^, and new strategies should be developed to understand carrier dynamics within semiconductors with single facets in the future.

In summary, BiOBr platelets exposing (001) reduction and (200) oxidation facets are employed as a model system to investigate the carrier transfer pathway modulated by built-in electric fields. The semiconductor/solution or semiconductor/metal-oxide nanoparticle interfaces provide variant IEFs in strength, which are all found to drive the directional migration of photo-excited electrons to the (001) facet with the similar modulation behavior that is characteristic of a Gaussian profile. Theoretical analysis validates the experimentally observed distribution profiles, which allow us to derive the drift distance steered by IEFs and the intrinsic diffusion length of electrons. This work lays a solid theoretical foundation and offers fundamental insights for understanding the charge transfer dynamics modulated by built-in IEFs in particulate photocatalysts. Based upon these findings, a significant enhancement of the photocatalytic performance of BiOBr platelets is achieved by matching particle sizes with the drift distances of electrons, unveiling the intrinsic structure-function relationships of photocatalysts.

## Methods

### Preparation of BiOBr platelets

All chemicals are commercially available and used as received. The typical procedure to prepare large BiOBr platelets (BiOBr-2.5): 1 mmol Bi(NO_3_)_3_·5H_2_O was added in 30 mL ethylene glycol (EG), and the solution was stirred for 30 min to completely dissolve (denoted as solution A). Subsequently, 3 mmol KBr was dissolved uniformly in 30 mL deionized water (marked as solution B). Then, solution A was transferred to a 50 mL syringe and injected into solution B (2 mL/min) with magnetic stirring, resulting in the formation of suspension. The resulting precipitate was harvested by centrifugation and washing with deionized water several times. Next, the samples were redispersed in 30 mL deionized water (pH = 1). The solution was sealed in a Teflon-lined autoclave (50 mL) and then hydrothermally treated at 140 °C for 24 h. After cooling to room temperature, the mixture was collected by centrifugation, washed with deionized water, ethanol and dried at 60 °C overnight. The synthesized BiOBr platelets have an average lateral size of about 2.5 μm and are denoted as BiOBr-2.5.

The synthesis method of BiOBr-0.5 platelets: 1 mmol Bi(NO_3_)_3_·5H_2_O was dispersed in 0.1 M mannitol solution (25 mL) with continued stirring for 30 min to achieve homogeneous dispersion. Then, 1 M KBr aqueous solution (5 mL) was slowly poured into the above-dispersed solution, resulting in a white precipitate. After stirring for 30 min, the resulting solution was heated at 160 °C for 3 h in a Teflon-lined autoclave (50 mL). The products were washed and dried by the same steps described above after cooling to room temperature. The obtained BiOBr platelets with a lateral size of 500 nm are denoted as BiOBr-0.5.

Preparation of BiOBr-0.5-B platelets: 1 mmol of Bi(NO_3_)_3_·5H_2_O was dissolved in ethylene glycol (30 mL, EG) for 30 min (denoted as solution A). Subsequently, 3 mmol KBr was uniformly dispersed in 30 mL of deionized water (marked as solution B). Then, solution A was transferred to a 50 mL syringe and injected into solution B with a drip rate of 2 mL/min, producing white suspension. After stirring for 10 h, the products were harvested by centrifugation and washing with deionized water several times. Next, the as-prepared sample was redispersed in 30 mL of deionized water (pH = 1) via ultrasound for 5 min. The mixture was sealed in a Teflon-lined autoclave (50 mL) and then hydrothermally treated at 120 °C for 1 h. The products were washed and dried by the same steps described above, and the lateral size of them is ca.500 nm (denoted as BiOBr-0.5-B).

The synthesis method of BiOBr-0.1 platelets: 5 mmol Bi(NO_3_)_3_·5H_2_O and 0.4 M mannitol were dispersed in 30 mL deionized water by magnetic stirring for 30 min (solution A). Subsequently, 10 mmol KBr was dissolved in aqueous solution B (30 mL). Then, at a rate of 2 mL/min, solution A was injected into solution B with continuous stirring. After that, the precipitate was harvested by centrifugation, washed with distilled water several times. Finally, the precipitate was dispersed in 30 mL deionized water (pH = 1), and sealed in a Teflon-lined autoclave (50 mL) for hydrothermal treatment at 140 °C for 24 h. The products were washed and dried by the same steps described above, and the lateral size of them is ca. 100 nm (denoted as BiOBr-0.1).

The synthesis of BiOBr-0.1-B platelets: 1 mL of oleic acid and 0.5 mL of oleylamine (as surfactants) were added into 15 mL of ethylene glycol (EG) dissolved with 1 mmol of Bi(NO_3_)_3_·5H_2_O (denoted as solution A). Subsequently, 3 mmol KBr was dissolved uniformly in 14 mL pH = 1 deionized water (marked as solution B). Then, solution A was transferred to a 50 mL syringe and injected into solution B (2 mL/min). After stirring for 2 h, the solution was sealed in a Teflon-lined autoclave (50 mL) and then hydrothermally treated at 140 °C for 1 h. The products were washed and dried by the same steps described above, and the lateral size of them is ca.100 nm (denoted as BiOBr-0.1-B).

The synthesis method of BiOBr platelets with an average lateral size of 50 nm (BiOBr-0.05): 5 mmol Bi(NO_3_)_3_·5H_2_O was added in 40 mL ethylene glycol (EG), and the mixture was immediately immersed in an ice-water bath for 30 min under continuous stirring (solution A). Then, 5 mmol KBr was dispersed in 40 mL distilled water and rapidly chilled in an ice-water bath for 30 min (solution B). After that, solution B was poured into solution A, and the mixed solution was stirred in an ice-water bath for 1 h, resulting in a white suspension. The samples were collected by the same steps described in the above procedures and named BiOBr-0.05.

### Photo-deposition of metal oxides and/or metals on BiOBr platelets

To photo-deposite CoO_x_ or MnO_x_ particles, 50 mg BiOBr powders and a certain content of Co(NO_3_)_2_·6H_2_O or Mn(NO_3_)_2_·4H_2_O (such as 1 wt.%) were mixed in distilled water (100 mL), and the mixture was then illuminated by a 300 W xenon lamp (an irradiation power density of 1.43 W/cm^2^) under full-spectrum irradiation for 30 min. After that, the suspension was harvested by filtration and washed with distilled water several times. The resulting sample was finally dried at 60 °C overnight.

For photo-reduction of Ag or Pt particles as probes to track photo-generated electrons. 50 mg of BiOBr powders were added in deionized water (100 mL) containing AgNO_3_ or H_2_PtCl_6_·6H_2_O as the precursor with an aqueous methanol solution (10 vol %), respectively. Subsequently, the suspension was illuminated by full-spectrum irradiation of a xenon lamp (300 W,) for 1 or 3 min to get the Ag/BiOBr or Pt/BiOBr pairs, respectively. In addition, Pt nanoparticles were photo-deposited on BiOBr platelets at different pH values (adjusted by dilute nitric acid).

We also investigated the effect of IEFs on the photo-reduction reactions. First, metal oxide co-catalysts (CoO_x_ or MnO_x_) were loaded onto BiOBr platelets to simulate a diversity of IEFs, using the above-mentioned photo-oxidation approach. Later, Ag, Pt, or Au particles were used as probes to track the dynamic migration of photo-excited electrons by using the precursors of AgNO_3_, H_2_PtCl_6_·6H_2_O, or HAuCl_4_·4H_2_O, following the same procedure described before. Those samples are named after the photo-deposition sequence, including CoO_x_-Ag/BiOBr, CoO_x_-Pt/BiOBr, CoO_x_-Au/BiOBr, MnO_x_-Ag/BiOBr, MnO_x_-Pt/BiOBr, MnO_x_-Au/BiOBr pairs.

For comparison, CoO_x_ and Ag co-catalysts were also loaded onto BiOBr platelets via a wet impregnation method. The Co(NO_3_)_2_·6H_2_O solution was injected into the solution of BiOBr platelets, stirred, and evaporated to dryness. Finally, the obtained powders were heated to 300 °C in a muffle furnace for 1 h. Similarly, the prepared Ag particles were also added into the solution of BiOBr platelets, stirred, and evaporated to dryness.

### The performance test of photocatalytic water oxidation reactions

The performance of photocatalytic water oxidation was evaluated in the presence of a glass closed gas circulation system with a Pyrex top-irradiation reaction vessel. In a typical case, 50 mg BiOBr photocatalysts and 1 mmol Fe(NO_3_)_3_·9H_2_O (as an electron acceptor) were added to 100 mL of distilled water. Then, the reaction system was evacuated under a vacuum pump to completely remove the air before irradiation with a 300 W xenon lamp (λ > 300 nm, an irradiation power density of 1.43 W/cm^2^). By circulating cooling water, the temperature of the photocatalytic reaction was maintained at room temperature. The evolved gases were collected and analyzed by online gas chromatography (Shimadzu, GC-8A) equipped with a thermal conductivity detector (TCD) and a molecular sieve column (5 Å) with Argon as the carrier gas.

### The performance test of photocatalytic reduction of Cr(VI) to Cr(III)

In a typical case, 20 mg photocatalyst was mixed with 80 mL Cr(VI) aqueous solution (40 ppm) in a 100 mL quartz reactor. Then, the solution was stirred for 30 min to reach adsorption-desorption equilibrium (under dark conditions). After that, the system was irradiated by a 300 W xenon lamp (λ > 300 nm, an irradiation power density of 1.43 W/cm^2^). During the illumination process, approximately 3 mL of suspension was taken from the reactor at a predetermined interval and a separate photocatalyst with a filter. The content of Cr(VI) in the supernatant solution was determined colorimetrically at 540 nm using the diphenylcarbazide method^65,66^. The measured absorbance intensities at different illumination times were transformed to the reduction ratio of Cr(VI), which is calculated using the following expression:

Reduction ratio of Cr(VI) = (C_0_-C_t_)/C_0_ × 100%

Where C_0_ and C_t_ are the absorbance intensities when illuminated for 0 and t min, respectively.

### Structure and composition characterization

NPowder X-ray diffraction (XRD) patterns were recorded on a Bruker D8 Advance diffractometer with Cu-Kα radiation (*λ* = 1.5406 Å). Scanning emission microscopy (SEM) measurements were carried out by using Hitachi SU-8010 Field emission SEM. Transmission electron microscopy (TEM) images, including bright field (BF), high-resolution electron microscopy (HREM), high-angle-annular dark field (HAADF) images, and energy dispersive spectrometry (EDS) maps were obtained on a Thermo Fisher Scientific TEM (Talos F200S). To measure the distances between the centers of metal particles and the edges of BiOBr platelets, BiOBr platelets were aligned along [001] zone axes in TEM to reduce measurement error arising from differences in view angles. UV/Vis diffuse reflectance spectroscopy (DRS) of the samples was measured on Hitachi UH 5300 spectrophotometer. To analyze the specific surface area of as-synthesized products, nitrogen adsorption-desorption isotherms were performed at 77 K using Micromeritics ASAP 2460 equipment. Electron energy loss spectroscopy (EELS) was carried out via an aberration-corrected transmission electron microscopy (Themis Z 3.2) working at 300 kV. The measured collection angle of EELS was 100 mrad.

### Photoluminescence characterization

The photoluminescence (PL) spectra and time-resolved measurements were collected using a Horiba Fluorolog-3 spectrophotometer.

### Photoelectrochemical measurements

Electrochemical measurements were conducted via a Biologic VSP-300 Electrochemical System in a conventional three-electrode cell, using a Pt plate (1 cm^2^) as the counter electrode and an Ag/AgCl electrode as the reference electrode with the 0.2 M Na_2_SO_4_ electrolyte. The active area is 0.25 cm^2^ for the working electrode. The working electrodes were coated with samples on fluorine-doped tin oxide (FTO) glass. FTO glass was masked using scotch tape, reserving an area of 0.25 cm^2^ to load BiOBr platelets. Then 5 mg BiOBr sample was dispersed in 1 mL of DMF by sonication to form a slurry. The slurry was spread onto the FTO glass. After air-drying, the scotch tape was removed, and the working electrode was further dried at 393 K for 2 h to improve adhesion. Finally, epoxy resin was coated to protect the sample-free region on the electrode. Chronoamperometry (CA) curve was conducted by recording the photocurrent with a light on and off with a 300 W xenon lamp (λ > 300 nm) under the potential of 0.2 V (versus Ag/AgCl). The electronic impedance spectra (EIS), Mott-Schottky (M-S), and Nyquist plots were measured in a 0.2 M Na_2_SO_4_ aqueous solution without any irradiation^67^.

### DFT calculation

The DFT calculations were performed using the projected augmented wave (PAW) method^68–70^, as implemented in the Vienna Ab-initio Simulation Package^54^. During structural relaxation, the generalized gradient approximation (GGA)^71^ with the Pardew-Burke-Brinkerhoff (PBE) scheme was used to describe the exchange-correlation function. The hybrid Heyd-Scuseria-Enrzerhof (HSE06) approximation to the exchange-correlation functional was used for accurate electronic structure calculations^55–58^. The kinetic energy cut-off of the plane wave basis set was set to 500 eV. The k-points were generated using the Monkhorst-Pack mesh^72^. The threshold for energy convergence for each iteration was set to 10^−6^ eV. Geometries were assumed to be converged when forces on each atom were less than 0.005 eV/Å. The Van der Waals (vdW) interactions were included using optB86b-vdW functionals^73^. The 9 × 9 × 4 k-point mesh was used for bulk BiOBr optimization. Both atomic positions and lattice constants were fully optimized during bulk calculation. The optimized lattice parameters of tetragonal BiOBr bulk are *a* = *b* = 3.94 Å and *c* = 8.13 Å, which are in very good agreement with the experimental results^74^. The slab models with a finite number of BiOBr sublayers were used for surface calculations. To avoid spurious interactions between periodic images, a vacuum layer of 15 Å thickness was added along the surface normal direction. The (001) and (200) surfaces of BiOBr were simulated by using seven and ten layers of BiOBr sublayers, and the k-point meshes of 9 × 9 × 1 and 4 × 10 × 1. During surface calculations, the lattice parameters were fixed and only atoms on the top layers were fully relaxed (Supplementary Fig. 26). Surface band structures were calculated along the special band path connecting the k-space high-symmetry points of G-M-X-G for the (001) surface, and G-X-S-Y-G for the (200) surface. Electrostatic potentials and work functions of the surface slab models were displayed in Supplementary Fig. 7.

## Supplementary information

Supplementary Information

## Data Availability

The authors declare that the data supporting the findings of this study are available in the paper and its supplementary information files. Source data are provided with this paper.

## Source data

Source Data

## References

[1] Fang Y (2020). Photocatalysis: an overview of recent developments and technological advancements. Sci. China.: Chem..

[2] Chen S, Takata T, Domen K (2017). Particulate photocatalysts for overall water splitting. Nat. Rev. Mater..

[3] Luo Z, Fang Y, Zhou M, Wang X (2019). A borocarbonitride ceramic aerogel for photoredox catalysis. Angew. Chem. Int. Ed..

[4] Hisatomi T, Kubota J, Domen K (2014). Recent advances in semiconductors for photocatalytic and photoelectrochemical water splitting. Chem. Soc. Rev..

[5] Takata T (2020). Photocatalytic water splitting with a quantum efficiency of almost unity. Nature.

[6] Khan K (2019). Spatial separation of dual-cocatalysts on one-dimensional semiconductors for photocatalytic hydrogen production. J. Mater. Chem. A.

[7] Qi M-Y (2020). Switching light for site-directed spatial loading of cocatalysts onto heterojunction photocatalysts with boosted redox catalysis. ACS Catal..

[8] Sun S, He L, Yang M, Cui J, Liang S (2022). Facet junction engineering for photocatalysis: A comprehensive review on elementary knowledge, facet-synergistic mechanisms, functional modifications, and future perspectives. Adv. Funct. Mater..

[9] Yu J, Low J, Xiao W, Zhou P, Jaroniec M (2014). Enhanced photocatalytic CO_2_-reduction activity of anatase TiO_2_ by coexposed {001} and {101} facets. J. Am. Chem. Soc..

[10] Zhang A-Y (2018). Epitaxial facet junctions on TiO_2_ single crystals for efficient photocatalytic water splitting. Energy Environ. Sci..

[11] Hao X (2019). Self-constructed facet junctions on hexagonal CdS single crystals with high photoactivity and photostability for water splitting. Appl. Catal. B: Environ..

[12] Ma Y, Wang X, Li C (2015). Charge separation promoted by phase junctions in photocatalysts. Chin. J. Catal..

[13] Chen F (2018). Thickness-dependent facet junction control of layered BiOIO_3_ single crystals for highly efficient CO_2_ photoreduction. Adv. Funct. Mater..

[14] Gao Y (2017). Directly probing charge separation at interface of TiO_2_ phase junction. J. Phys. Chem. Lett..

[15] Li J, Zhan G, Yu Y, Zhang L (2016). Superior visible light hydrogen evolution of Janus bilayer junctions via atomic-level charge flow steering. Nat. Commun..

[16] Zhang J, Zhang M, Sun R-Q, Wang X (2012). A facile band alignment of polymeric carbon nitride semiconductors to construct isotype heterojunctions. Angew. Chem. Int. Ed..

[17] Duan X (2014). Lateral epitaxial growth of two-dimensional layered semiconductor heterojunctions. Nat. Nanotechnol..

[18] Xue S (2022). Interfacial engineering of lattice coherency at ZnO-ZnS photocatalytic heterojunctions. Chem. Catal..

[19] Guan M (2013). Vacancy associates promoting solar-driven photocatalytic activity of ultrathin bismuth oxychloride nanosheets. J. Am. Chem. Soc..

[20] Li R (2013). Spatial separation of photogenerated electrons and holes among {010} and {110} crystal facets of BiVO_4_. Nat. Commun..

[21] Zhang D, Li J, Wang Q, Wu Q (2013). High {001} facets dominated BiOBr lamellas: Facile hydrolysis preparation and selective visible-light photocatalytic activity. J. Mater. Chem. A.

[22] Wu X (2017). Improving the photo-oxidative capability of BiOBr via crystal facet engineering. J. Mater. Chem. A.

[23] Mu L (2016). Enhancing charge separation on high symmetry SrTiO_3_ exposed with anisotropic facets for photocatalytic water splitting. Energy Environ. Sci..

[24] Bai S (2015). Toward enhanced photocatalytic oxygen evolution: Synergetic utilization of plasmonic effect and schottky junction via interfacing facet selection. Adv. Mater..

[25] Li L, Salvador PA, Rohrer GS (2014). Photocatalysts with internal electric fields. Nanoscale.

[26] Liu Y (2020). Internal-field-enhanced charge separation in a single-domain ferroelectric PbTiO_3_ photocatalyst. Adv. Mater..

[27] Deng Y (2022). Spatial separation of photogenerated charges on well-defined bismuth vanadate square nanocrystals. Small.

[28] Li Z (2020). Surface-polarity-induced spatial charge separation boosts photocatalytic overall water splitting on GaN nanorod arrays. Angew. Chem. Int. Ed..

[29] Bai Y (2019). Homophase junction for promoting spatial charge separation in photocatalytic water splitting. ACS Catal..

[30] Guo Y, Shi W, Zhu Y (2019). Internal electric field engineering for steering photogenerated charge separation and enhancing photoactivity. EcoMat.

[31] Stranks SD (2013). Electron-hole diffusion lengths exceeding 1 micrometer in an organometal trihalide perovskite absorber. Science.

[32] Shaw PE, Ruseckas A, Samuel IDW (2008). Exciton diffusion measurements in Poly(3-hexylthiophene). Adv. Mater..

[33] Dey A (2016). Light induced charge transport property analysis of nanostructured ZnS based Schottky diode. J. Mater. Sci: Mater. Electron.

[34] Lin JDA (2014). Systematic study of exciton diffusion length in organic semiconductors by six experimental methods. Mater. Horiz..

[35] Mikhnenko OV, Blom PWM, Nguyen T-Q (2015). Exciton diffusion in organic semiconductors. Energy Environ. Sci..

[36] Tyagi MS, Van Overstraeten R (1983). Minority carrier recombination in heavily-doped silicon. Solid-State Electron.

[37] del Alamo JA, Swanson RM (1987). Modelling of minority-carrier transport in heavily doped silicon emitters. Solid-State Electron.

[38] Nozik AJ, Memming R (1996). Physical chemistry of semiconductor−liquid interfaces. J. Phys. Chem..

[39] Sawada A (2006). Internal electric fields of electrolytic solutions induced by space-charge polarization. J. Appl. Phys..

[40] Chen R, Zhu J, An H, Fan F, Li C (2017). Unravelling charge separation via surface built-in electric fields within single particulate photocatalysts. Faraday Discuss.

[41] Li J, Cai L, Shang J, Yu Y, Zhang L (2016). Giant enhancement of internal electric field boosting bulk charge separation for photocatalysis. Adv. Mater..

[42] Zhu J (2015). Direct imaging of highly anisotropic photogenerated charge separations on different facets of a single BiVO_4_ photocatalyst. Angew. Chem. Int. Ed..

[43] Mu L, Zeng B, Tao X, Zhao Y, Li C (2019). Unusual charge distribution on the facet of a SrTiO_3_ nanocube under light irradiation. J. Phys. Chem. Lett..

[44] Ye L, Su Y, Jin X, Xie H, Zhang C (2014). Recent advances in BiOX (X = Cl, Br and I) photocatalysts: Synthesis, modification, facet effects and mechanisms. Environ. Sci.: Nano.

[45] Cheng H, Huang B, Dai Y (2014). Engineering BiOX (X = Cl, Br, I) nanostructures for highly efficient photocatalytic applications. Nanoscale.

[46] Yang Y (2018). BiOX (X=Cl, Br, I) photocatalytic nanomaterials: Applications for fuels and environmental management. Adv. Colloid Interface Sci..

[47] Li J, Yu Y, Zhang L (2014). Bismuth oxyhalide nanomaterials: Layered structures meet photocatalysis. Nanoscale.

[48] Ganose AM, Cuff M, Butler KT, Walsh A, Scanlon DO (2016). Interplay of orbital and relativistic effects in bismuth oxyhalides: BiOF, BiOCl, BiOBr, and BiOI. Chem. Mater..

[49] Li M (2019). Unprecedented eighteen-faceted BiOCl with a ternary facet junction boosting cascade charge flow and photo-redox. Angew. Chem. Int. Ed..

[50] Shi M (2020). Intrinsic facet-dependent reactivity of well-defined BiOBr nanosheets on photocatalytic water splitting. Angew. Chem. Int. Ed..

[51] Parrott JE (1985). The theory of majority-carrier motion in the Haynes–Shockley experiment. Solid-State Electron.

[52] Shacham‐Diamand Y, Kidron I (1984). Haynes-Shockley experiment on n-type HgCdTe. J. Appl. Phys..

[53] Haynes JR, Shockley W (1949). Investigation of hole injection in transistor action. Phys. Rev..

[54] Kresse G, Furthmüller J (1996). Efficient iterative schemes for ab initio total-energy calculations using a plane-wave basis set. Phys. Rev. B.

[55] Paier J (2006). Screened hybrid density functionals applied to solids. J. Chem. Phys..

[56] Heyd J, Scuseria GE (2004). Efficient hybrid density functional calculations in solids: Assessment of the Heyd–Scuseria–Ernzerhof screened Coulomb hybrid functional. J. Chem. Phys..

[57] Heyd J, Scuseria GE, Ernzerhof M (2003). Hybrid functionals based on a screened Coulomb potential. J. Chem. Phys..

[58] Yu J, Li T, Sun Q (2019). Single-layer BiOBr: An effective p-type 2D thermoelectric material. J. Appl. Phys..

[59] Malis T, Cheng SC, Egerton RF (1988). EELS log-ratio technique for specimen-thickness measurement in the TEM. J. Electron Microsc. Tech..

[60] Lee C-W, Ikematsu Y, Shindo D (2000). Thickness measurement of amorphous SiO_2_ by EELS and electron holography. Mater. Trans., JIM.

[61] Heo Y-U (2020). Comparative study on the specimen thickness measurement using EELS and CBED methods. Appl. Microsc.

[62] Amaechi IC, Katoch R, Kolhatkar G, Sun S, Ruediger A (2020). Particle size effect on the photocatalytic kinetics of barium titanate powders. Catal. Sci. Technol..

[63] Han L, Guo Y, Lin Z, Huang H (2020). 0D to 3D controllable nanostructures of BiOBr via a facile and fast room-temperature strategy. Colloid Surf. A.

[64] Rothenberger G, Moser J, Graetzel M, Serpone N, Sharma DK (1985). Charge carrier trapping and recombination dynamics in small semiconductor particles. J. Am. Chem. Soc..

[65] Shen L, Liang S, Wu W, Liang R, Wu L (2013). Multifunctional NH_2_-mediated zirconium metal–organic framework as an efficient visible-light-driven photocatalyst for selective oxidation of alcohols and reduction of aqueous Cr(VI). Dalton Trans..

[66] Idris A, Hassan N, Rashid R, Ngomsik A-F (2011). Kinetic and regeneration studies of photocatalytic magnetic separable beads for chromium (VI) reduction under sunlight. J. Hazard. Mater..

[67] Ou H, Chen X, Lin L, Fang Y, Wang X (2018). Biomimetic donor–acceptor motifs in conjugated polymers for promoting exciton splitting and charge separation. Angew. Chem. Int. Ed..

[68] Blöchl PE (1994). Projector augmented-wave method. Phys. Rev. B.

[69] Kresse G, Joubert D (1999). From ultrasoft pseudopotentials to the projector augmented-wave method. Phys. Rev. B.

[70] Hohenberg P, Kohn W (1964). Inhomogeneous electron gas. Phys. Rev..

[71] Perdew JP, Burke K, Ernzerhof M (1996). Generalized gradient approximation made simple. Phys. Rev. Lett..

[72] Monkhorst HJ, Pack JD (1976). Special points for Brillouin-zone integrations. Phys. Rev. B.

[73] Klimeš J, Bowler DR, Michaelides A (2009). Chemical accuracy for the van der Waals density functional. J. Phys.: Condens. Matter.

[74] Zhang X, Ai Z, Jia F, Zhang L (2008). Generalized one-pot synthesis, characterization, and photocatalytic activity of hierarchical BiOX (X = Cl, Br, I) nanoplate microspheres. J. Phys. Chem. C..

