# Supplementary material for: Unveiling the charge transfer dynamics steered by built-in electric fields in BiOBr photocatalysts
Source: Nat Commun. 2022 Apr 25;13:2230. doi: 10.1038/s41467-022-29825-0 (PMC9038904; doi:10.1038/s41467-022-29825-0)
Supplement: Supplementary file 1 — Supplementary Information [file 41467_2022_29825_MOESM1_ESM.pdf]

Supplementary Information for

Unveiling the Charge Transfer Dynamics Steered by Built-in Electric Fields in BiOBr Photocatalysts

Zhishan Luo[1,2], Xiaoyuan Ye[1], Shijia Zhang[1], Sikang Xue[1], Can Yang[1], Yidong Hou[1], Wandong Xing[3], Rong Yu[3], Jie Sun[4], Zhiyang Yu[1] and Xinchen Wang*[1]*

1. State Key Laboratory of Photocatalysis on Energy and Environment, College of Chemistry, Fuzhou University, Fuzhou Fujian 350108, P. R. China.
2. College of Chemical Engineering, Fuzhou University, Fuzhou, Fujian 350108, P. R. China.
3. National Center for Electron Microscopy in Beijing, School of Materials Science and Engineering, Key Laboratory of Advanced Materials of Ministry of Education of China, State Key Laboratory of New Ceramics and Fine Processing, Tsinghua University, Beijing, 100084, P. R. China.
4. Fujian Science & Technology Innovation Laboratory for Optoelectronic Information of China, Fuzhou 350100, China and College of Physics and Information Engineering, Fuzhou University, Fuzhou 350100, China.

*To whom correspondence should be addressed.

Email: yuzyemlab@fzu.edu.cn; xcwang@fzu.edu.cn

Website: http://wanglab.fzu.edu.cn

Contents

Contents ························ 2

Supplementary Figures ························ 4

Supplementary Figure 1. Morphology of BiOBr-2.5 platelets. ························ 4

Supplementary Figure 2. Photo-deposition of CoO_x nanoparticles on the surfaces of BiOBr platelets. ························ 5

Supplementary Figure 3. Loading CoO_x nanoparticles on the surfaces of BiOBr platelets by a wet impregnation method. ························ 6

Supplementary Figure 4. Loading Ag nanoparticles on the surfaces of BiOBr platelets by a wet impregnation method. ························ 7

Supplementary Figure 5. Collective charge transfer pathway steered by IEFs for MnO_x-metal(Ag, Pt, Au)/BiOBr photocatalysts. ························ 8

Supplementary Figure 6. Collective charge transfer pathway steered by IEFs with different pH values for Pt/BiOBr photocatalysts. ························ 9

Supplementary Figure 7. DFT calculations for the band structures of BiOBr photocatalysts. ························ 10

Supplementary Figure 8. Facet junctions within BiOBr platelets. ························ 11

Supplementary Figure 9. Schematic illustration of the carrier transfer pathway of photo-generated electrons within BiOBr platelets. ························ 12

Supplementary Figure 10. Numeral simulation of carrier transfer within BiOBr platelets. ·· 13

Supplementary Figure 11. Thickness measurement for BiOBr platelets by an electron energy loss spectroscopy (EELS) technique. ························ 14

Supplementary Figure 12. Thickness versus carrier transport parameters in four individual BiOBr platelets. ························ 15

Supplementary Figure 13. The relationship between thickness and charge transfer dynamics for every single BiOBr platelet. ························ 16

Supplementary Figure 14. Loading Ag nanoparticles on the surfaces of BiOCl platelets by photo-deposition. ························ 17

Supplementary Figure 15. BiOBr platelets are produced by different synthetical methods with varied lateral sizes. ························ 18

Supplementary Figure 16. DRS spectra of BiOBr-2.5, -0.5, -0.1 and -0.05 photocatalysts. · 19

Supplementary Figure 17. Impact of synthetical methods on the chemical, physical and optical properties of BiOBr platelets. ························ 20

Supplementary Figure 18. Charge transfer dynamics of BiOBr platelets produced by different synthesis methods and their photocatalytic performance. The thickness of BiOBr platelets is fixed to 0.1 and 0.5 μm. ········ 21

Supplementary Figure 19. The effect of sample thickness on the charge transfer kinetics. ·· 22

Supplementary Figure 20. Photo-deposition of Ag nanoparticles on the surfaces of BiOBr platelets when their thickness are reduced to ~50 nm. ········ 23

Supplementary Figure 21. Time-resolved lifetime measurements of BiOBr-2.5, -0.5, -0.1 and -0.05 photocatalysts. ········ 24

Supplementary Figure 22. Electrochemical impedance spectroscopy (EIS) spectra of BiOBr-2.5, -0.5, -0.1 and -0.05 platelets. ········ 25

Supplementary Figure 23. Photocatalytic performance of BiOBr-0.1 platelets under different pH values conditions. ········ 26

Supplementary Figure 24. Collective charge transfer migration steered by IEFs for $BiVO_4$ photocatalysts. ········ 27

Supplementary Figure 25. Photo-deposition of Ag nanoparticles on the surfaces of some common photocatalysts. ········ 28

Supplementary Figure 26. Atomic models of (001) and (200) surfaces of BiOBr photocatalysts. ········ 29

Supplementary Table ········ 30

Supplementary Table 1. A summary of the lateral drift distances and the diffusion lengths of electrons using MnO_x nanoparticles or solutions with varying pH values to construct lateral IEFs. ········ 30

Supplementary References ········ 31

Supplementary Figures

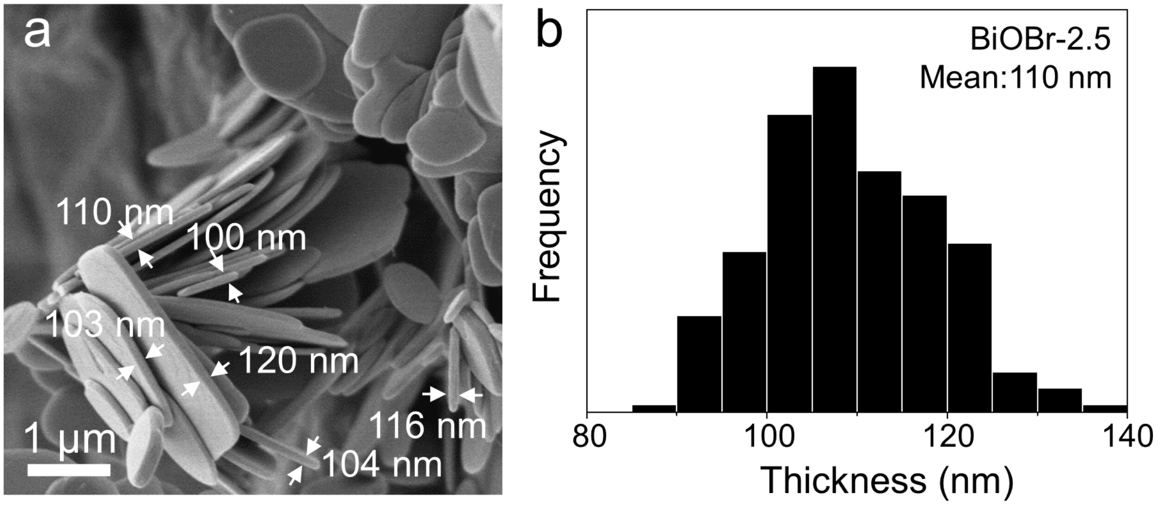

Supplementary Figure 1. Morphology of BiOBr-2.5 platelets.
(**a**) SEM image and (**b**) thickness statistics of BiOBr-2.5 platelets. Source data are provided as a Source Data file.

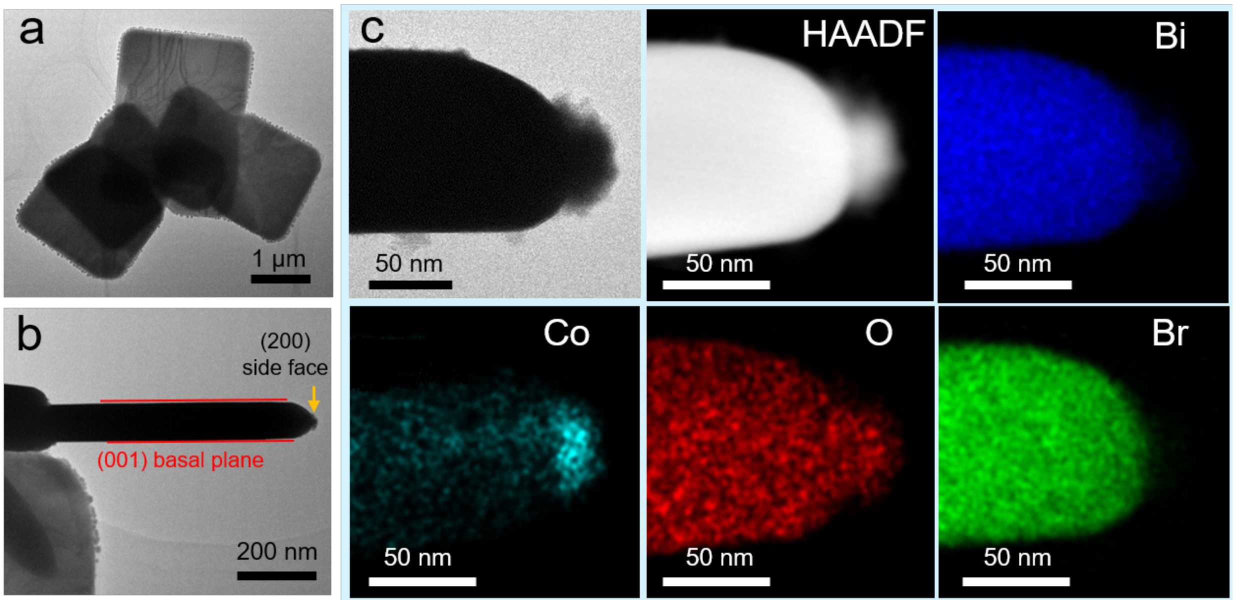

Supplementary Figure 2. Photo-deposition of CoO_x nanoparticles on the surfaces of BiOBr platelets.
(**a**) Top-view TEM image, (**b**) Side-view TEM image, and (**c**) Side-view TEM, HAADF images, and EDS maps showing the selective photo-deposition of CoO_x nanoparticles on the {200} side faces of BiOBr platelets.

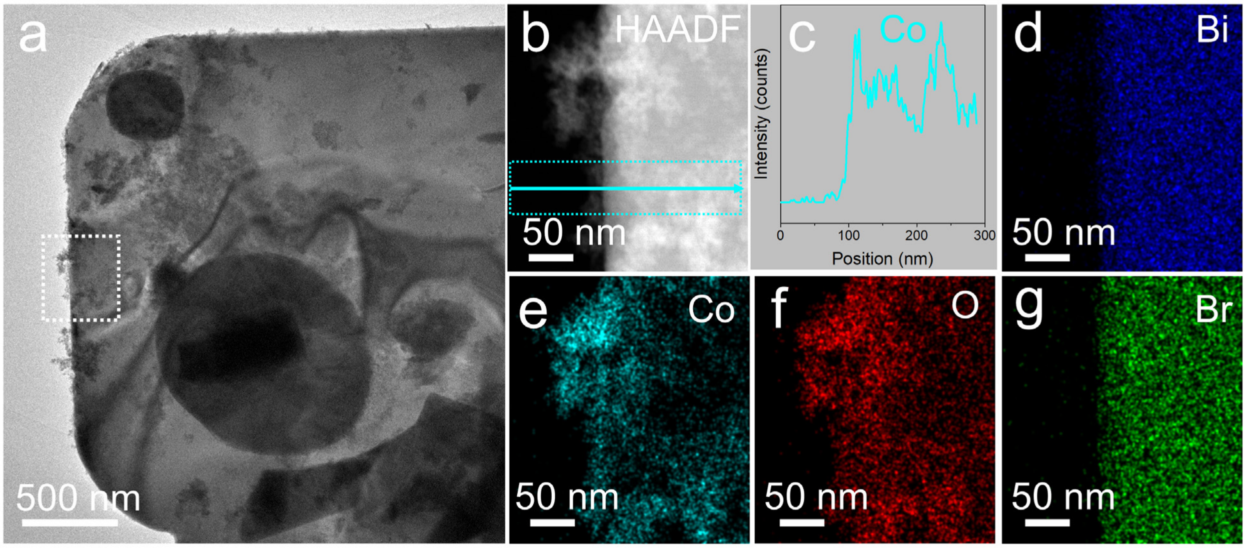

Supplementary Figure 3. Loading CoO_x nanoparticles on the surfaces of BiOBr platelets by a wet impregnation method.

(**a**) TEM, (**b**) HAADF, (**c**) HAADF line profile, and (**d-g**) EDS maps of BiOBr platelets prepared by a wet impregnation method. CoO_x particles are deposited on the side faces and basal facets of BiOBr platelets. Source data are provided as a Source Data file.

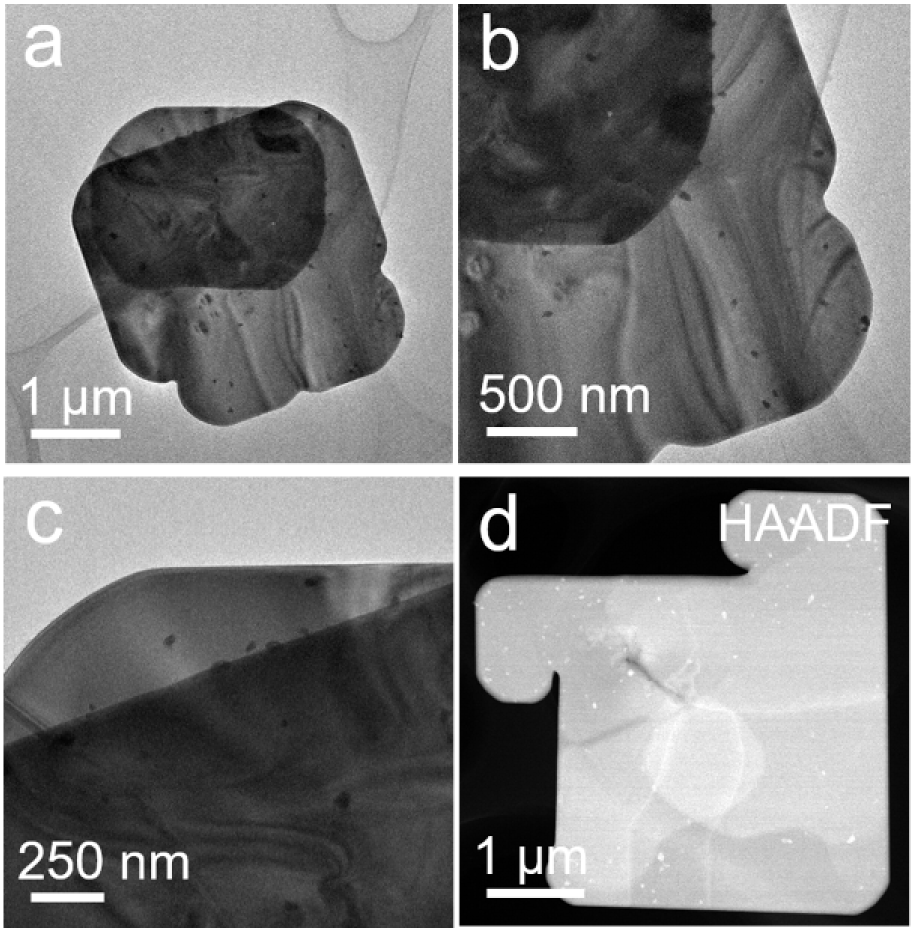

Supplementary Figure 4. Loading Ag nanoparticles on the surfaces of BiOBr platelets by a wet impregnation method.
(**a**-**c**) TEM images and (**d**) HAADF image showing randomly distributed Ag nanoparticles on the basal facets of BiOBr platelets prepared by a wet impregnation method.

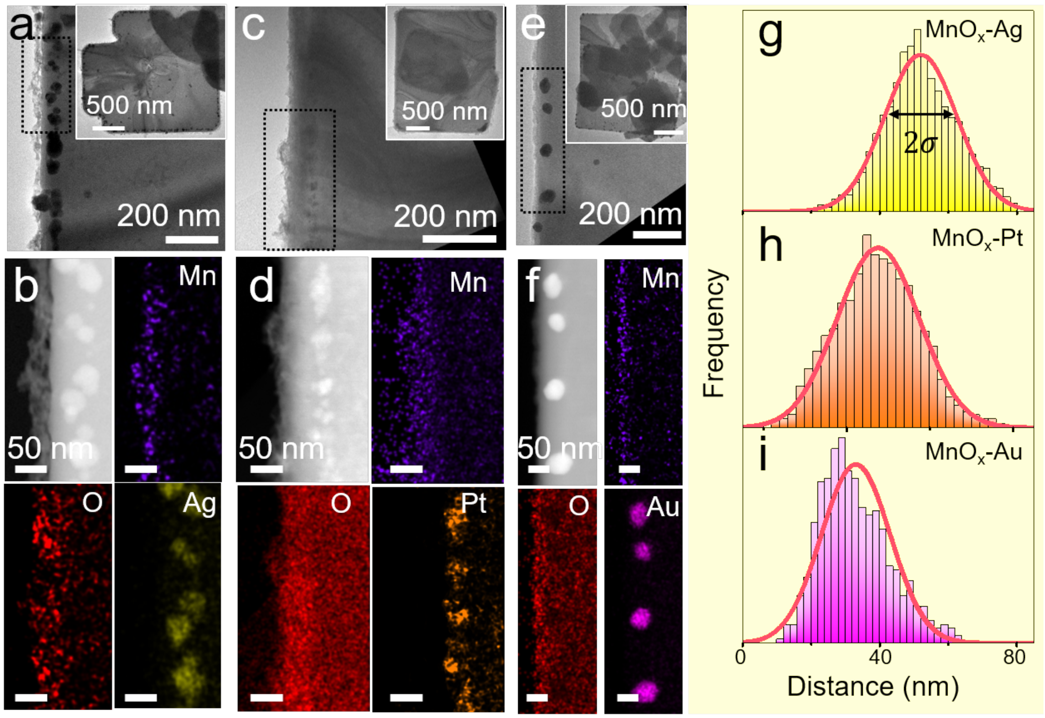

Supplementary Figure 5. Collective charge transfer pathway steered by IEFs for MnO_x-metal(Ag, Pt, Au)/BiOBr photocatalysts.

(**a**) TEM images and (**b**) EDS maps acquired after loading dual co-catalysts in the order of MnO_x and Ag nanoparticles. The MnO_x-Pt and MnO_x-Au pairs are shown in (**c-d**) and (**e-f**), respectively. Histogram diagrams of the distance from the centers of metal nanoparticles to the platelet edges are plotted in (**g-i**). Source data are provided as a Source Data file.

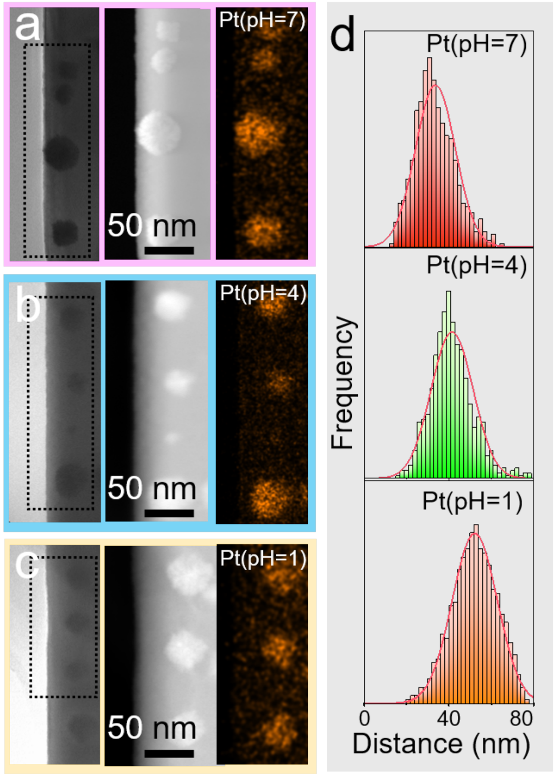

Supplementary Figure 6. Collective charge transfer pathway steered by IEFs with different pH values for Pt/BiOBr photocatalysts.

TEM, HAADF images, and EDS maps show the spatial distribution of photo-deposited Pt nanoparticles at varied pH values: (**a**) pH=7, (**b**) pH=4, and (**c**) pH=1. (**d**) Statistical histograms of the distances from the centers of Pt particles to the BiOBr platelet edges as a function of pH. Source data are provided as a Source Data file.

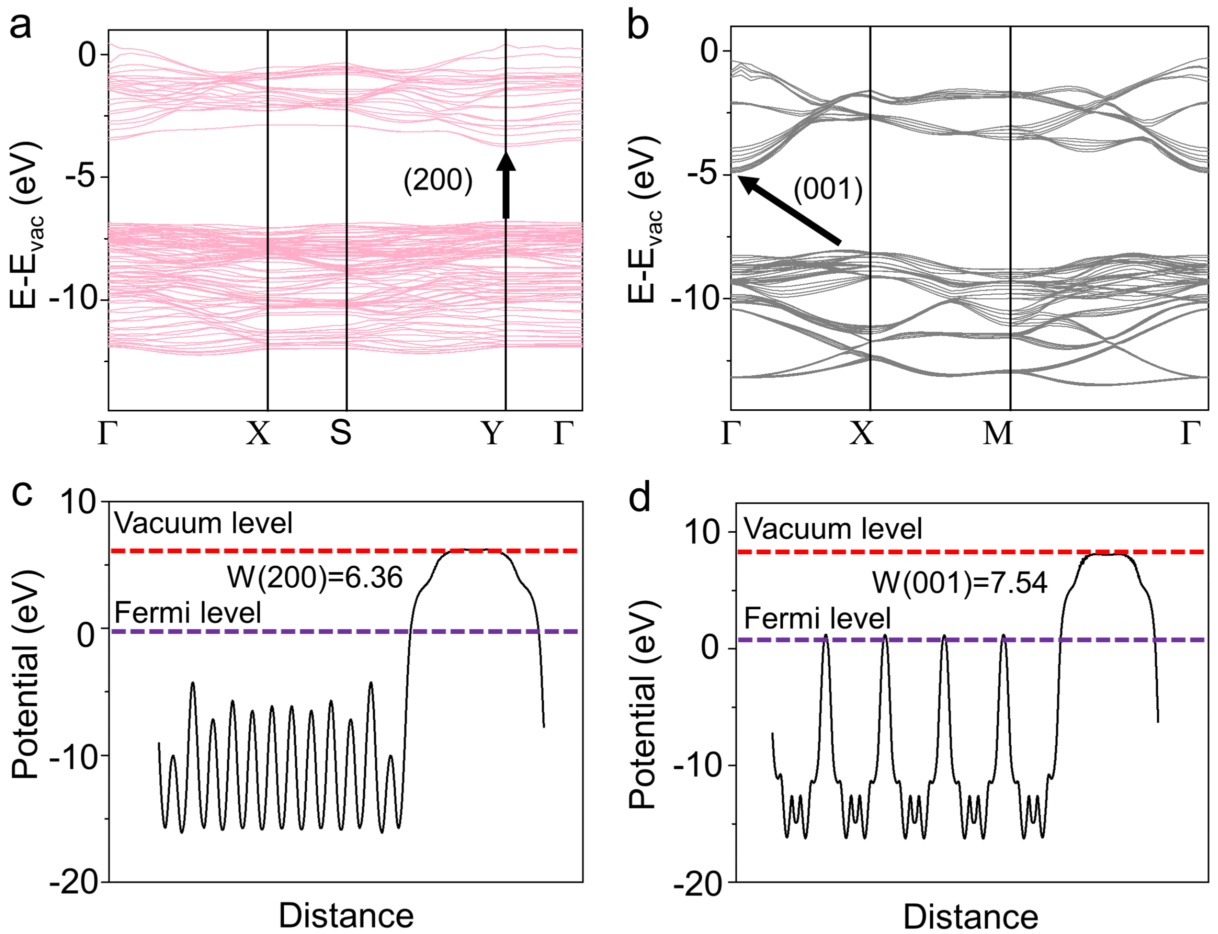

Supplementary Figure 7. DFT calculations for the band structures of BiOBr photocatalysts. DFT calculated band structure of the (**a**) (200) and (**b**) (001) facets of BiOBr photocatalysts. The electrostatic potentials and work functions of (**c**) (200) and (**d**) (001) surface slab models. Source data are provided as a Source Data file.

Based on the calculated electronic band structures of the {200} and {001} facets in Fig. 4a and Supplementary Fig. 7a,b, the recalculated band gaps of the {200} and {001} surfaces are 3.05 and 3.11 eV, respectively. The DFT calculations of work function for the {200} and {001} facets are plotted in Supplementary Fig. 7c,d, the conduction band offset (CBO) and valence band offset (VBO) are 1.18 and 1.24 eV, respectively[1, 2]. Hence, as shown in Fig. 4b, the photo-generated electrons are predominantly migrated to {001} basal planes, and holes prefer to accumulate on the lateral facets of {200} after the Fermi energies achieve the same level.

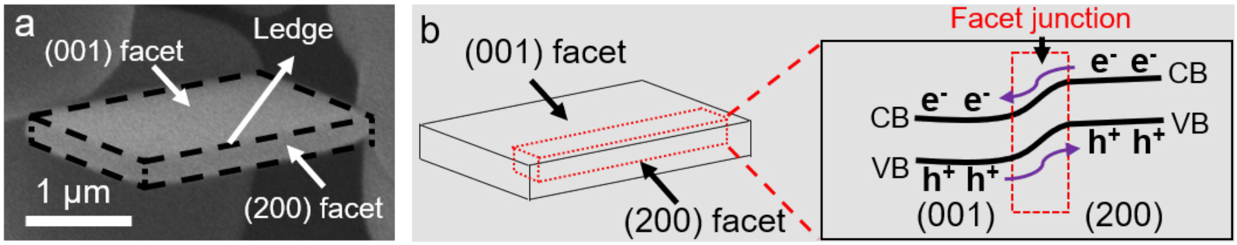

Supplementary Figure 8. Facet junctions within BiOBr platelets.
(**a**) SEM image of a crystal BiOBr platelet. **(b)** The schematic diagram of facet junctions for BiOBr platelets and the possible transfer pathway of charge carriers on a (200)/(001) facet junction.

The facet junction in our case differs from a semiconductor/semiconductor interface in physical, while it plays a similar charge carrier separation role. Here, single-crystalline BiOBr semiconductors in a form of platelet are produced by co-exposing {200} and {001} facets (Supplementary Fig. 8a). Facet junctions are readily formed at the ledge between {200} and {001} facets (as illustrated by black dashed lines in Supplementary Fig. 8a). It is defined as a homojunction between neighboring facets in a single-crystalline semiconductor, where continuous band bending and efficient charge separation occurs (right panel of Supplementary Fig. 8b)[3-5].

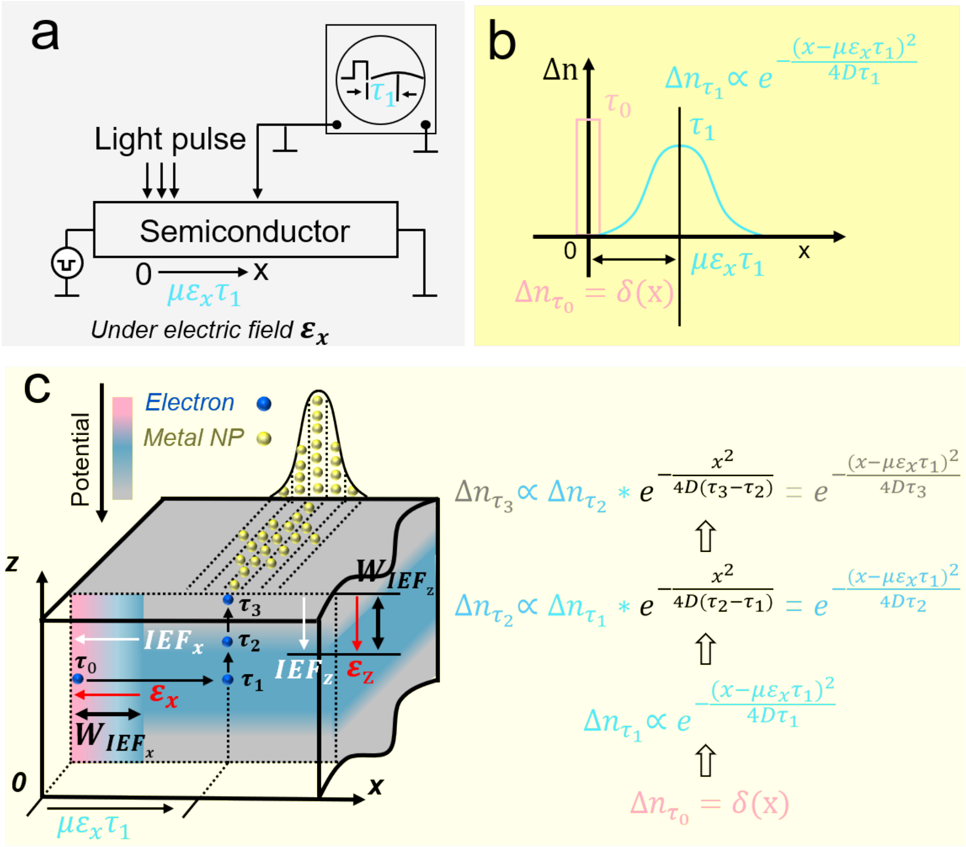

Supplementary Figure 9. Schematic illustration of the carrier transfer pathway of photo-generated electrons within BiOBr platelets.
(**a**) Experimental setup used to measure the drift mobility of electrons in the Haynes-Shockley experiment[6, 7]. (**b**) The generation of pulse induced electrons (τ_0) gives a Dirac delta function. Equation (1) is the continuity equation that describes a change in carrier density over time due to a difference between the incoming and outgoing flux of carriers as well as the recombination. After applying a uniform electric field, an electric term will show up in Equation (2) as the third term. The solution is derived in Equation (3), which indicates that 1) the non-equilibrium carrier will travel with a drift (group) velocity that is proportional to the external electric field and 2) the spatial distribution follows a characteristic Gaussian function as dictated by the $\exp\left[-\frac{(x-\mu_n\varepsilon t)^2}{4D_n t}\right]$ term. The modulating effect of external electric field to non-equilibrium carrier dynamic is plotted in (**b**). (**c**) A sketch describes the spatial distribution evolution of electrons in a BiOBr platelet with a (001)/(200) facet junction. The electron distribution evolution over time is derived in the right panel.

$$\frac{\partial \Delta n}{\partial t} = D_n \frac{\partial^2 \Delta n}{\partial x^2} - \frac{\Delta n}{\tau_n} \tag{1}$$

$$\frac{\partial \Delta n}{\partial t} = D_n \frac{\partial^2 \Delta n}{\partial x^2} - \frac{\Delta n}{\tau_n} - \mu_n \varepsilon \frac{\partial \Delta n}{\partial t} \tag{2}$$

$$\Delta n = \frac{N_n}{\sqrt{4\pi D_n t}} \exp\left[-\frac{(x-\mu_n\varepsilon t)^2}{4D_n t}\right] \exp\left[-\frac{t}{\tau}\right] \tag{3}$$

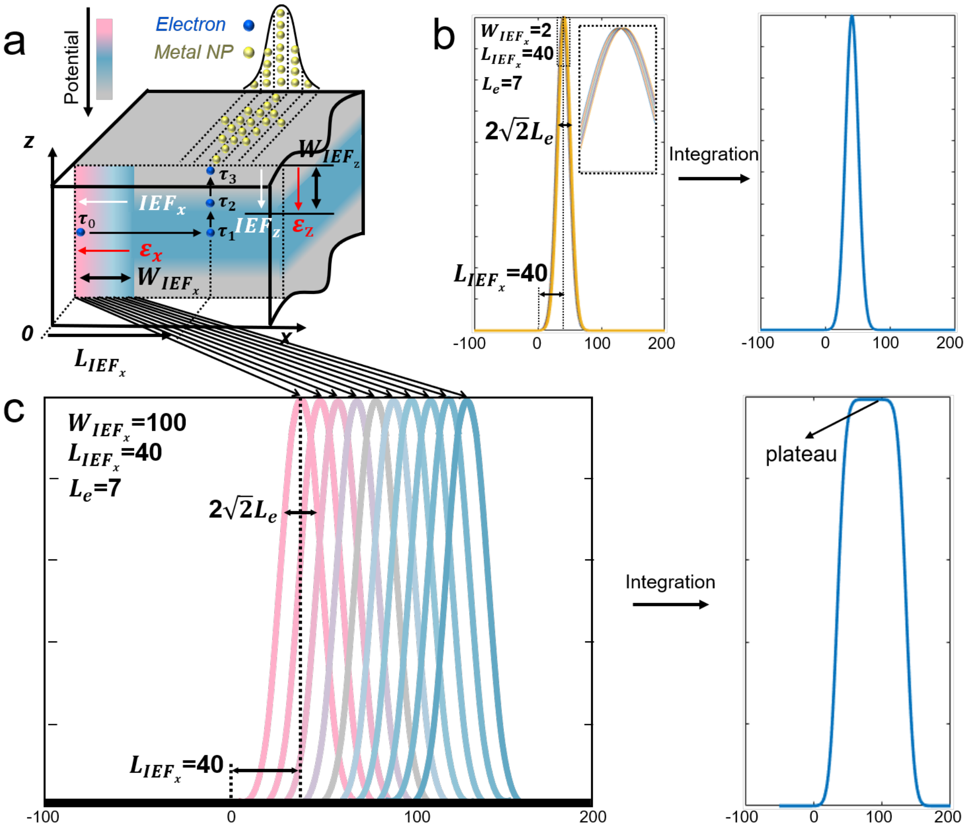

Supplementary Figure 10. Numeral simulation of carrier transfer within BiOBr platelets. The effect of the width of lateral IEF on the spatial distribution of electron arriving at the reduction (001) basal facet. (**a**) A schematic diagram showing the generation of electrons in the lateral space charge region. To simulate the effect of the width of lateral IEF (IEF_x) numerically, the space charge region is evenly divided into ten zones. First, a series of pulse signals are produced. Then, all the pulse signals drift by an identical length (L_{IEF_x}). In the end, the integration of all these signals corresponds to the distribution of drifted electrons. (**b**) W_{IEF_x}=2, L_{IEF_x}=40 and L_e=7 for a small W_{IEF_x} case. The integrated profile exhibits a Gaussian curve. (**c**) W_{IEF_x}=100, L_{IEF_x}=40 and L_e=7. It shows that, for a large W_{IEF_x} case, a plateau feature is convoluted in the profile.

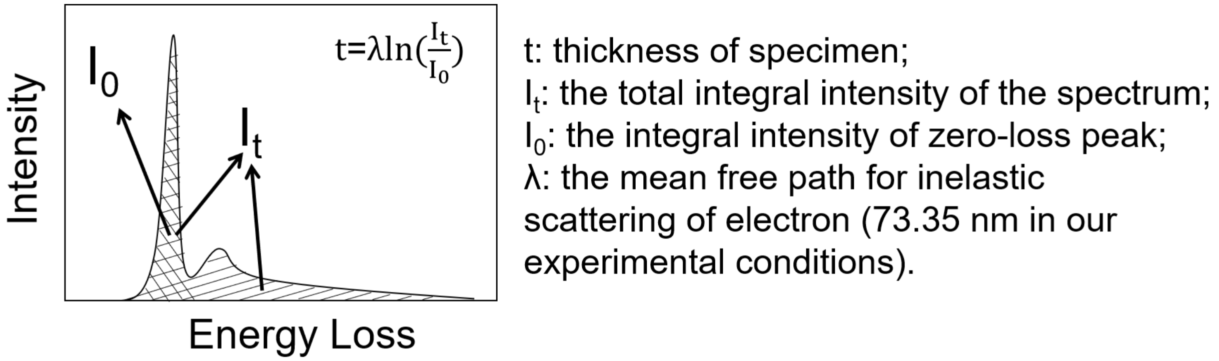

Supplementary Figure 11. Thickness measurement for BiOBr platelets by an electron energy loss spectroscopy (EELS) technique.
The specimen thickness could be calculated using the formula in the inset[8]. The mean free path could be estimated using numerical calculations in the literatures[9-11].

In order to uncover how changes in the thickness of BiOBr photocatalysts affect its charge dynamic, we correlated the spatial distribution of Ag nanoparticles to the platelet thickness (using an electron energy loss spectroscopy (EELS) technique (Supplementary Fig. 11))[8-11] for a same BiOBr platelet. Overall, we have measured four BiOBr-2.5 platelets. Thereby, a relationship between its charge dynamics and its corresponding sample thickness could be established.

The principle of the measuring method is based on the reference (Supplementary Fig. 11)[8-11]. The total integral intensity of the spectrum (I_t) and the integral intensity of zero-loss peak ((I_0)) are connected with the thickness (t) of BiOBr platelets, in the following formula: $t = \lambda \ln\left(\frac{I_t}{I_0}\right)$, where λ is the mean free path for inelastic scattering of the electron and is 73.35 nm in our experimental conditions. Therefore, we can get the value of $\ln\left(\frac{I_t}{I_0}\right)$ from the EELS spectra and further obtain the thickness of BiOBr platelets based on the above equation.

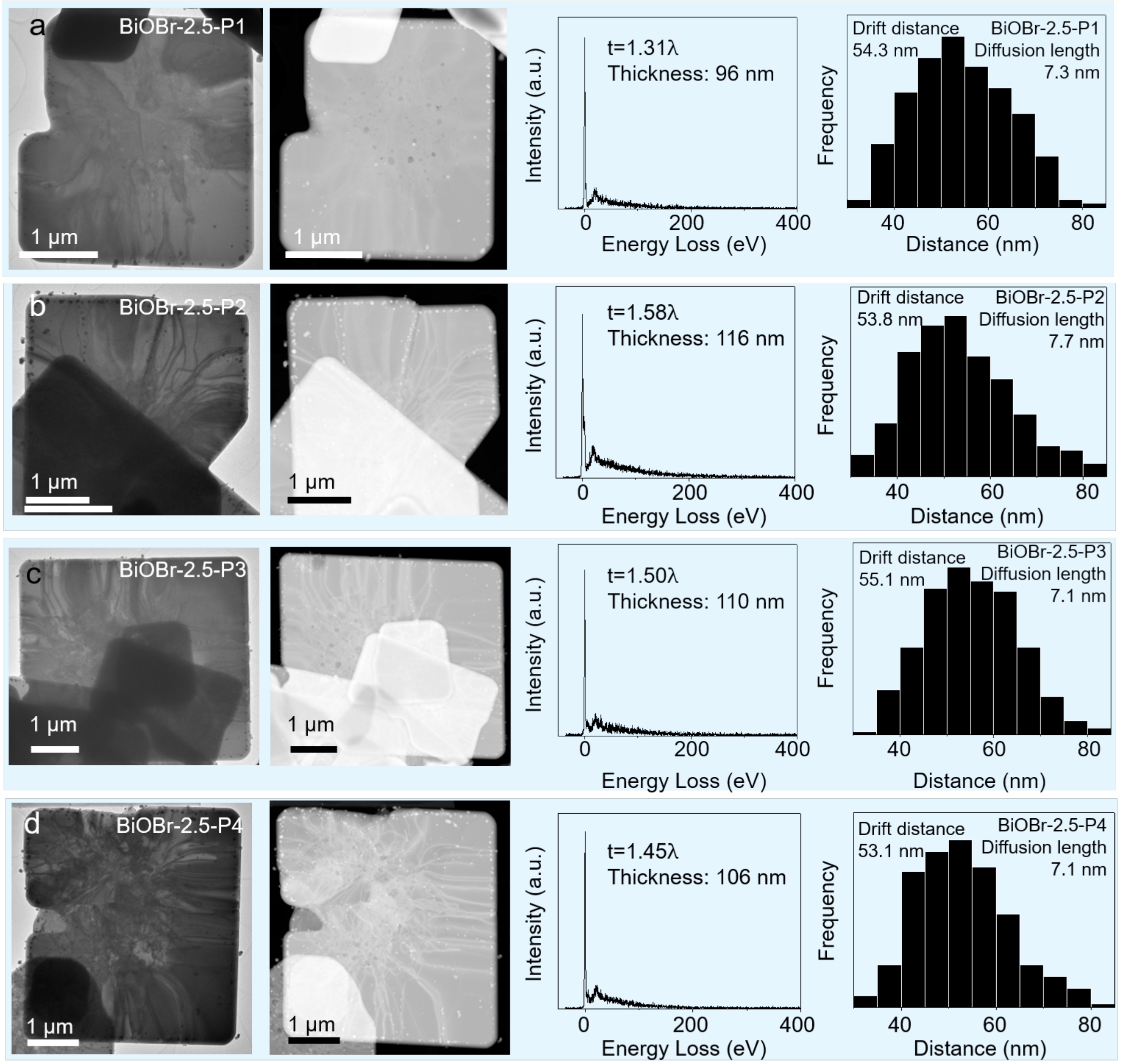

Supplementary Figure 12. Thickness versus carrier transport parameters in four individual BiOBr platelets.
TEM, HAADF images, electron energy-loss spectra, and the corresponding statistical histograms of the distances from the centers of Ag nanoparticles to the edges of BiOBr platelets for (**a**) BiOBr-2.5-P1, (**b**) BiOBr-2.5-P2, (**c**) BiOBr-2.5-P3, and (**d**) BiOBr-2.5-P4 photocatalysts. Source data are provided as a Source Data file.

Four BiOBr-2.5 platelets (denoted as BiOBr-2.5-P1, BiOBr-2.5-P2, BiOBr-2.5-P3, and BiOBr-2.5-P4 platelets) are measured, and their EELS spectra are plotted in Supplementary Fig. 12. From the above results of spectra, the thickness of BiOBr-2.5-P1, BiOBr-2.5-P2, BiOBr-2.5-P3, and BiOBr-2.5-P4 platelets are 96, 116, 110 and 106 nm, respectively (Supplementary Fig. 12). After statistical analysis, the drift distance/diffusion length of BiOBr-2.5-P1, BiOBr-2.5-P2, BiOBr-2.5-P3, and BiOBr-2.5-P4 platelets are 54.3/7.3, 53.8/7.7, 55.1/7.1 and 53.1/7.1 nm, respectively (Supplementary Fig. 12).

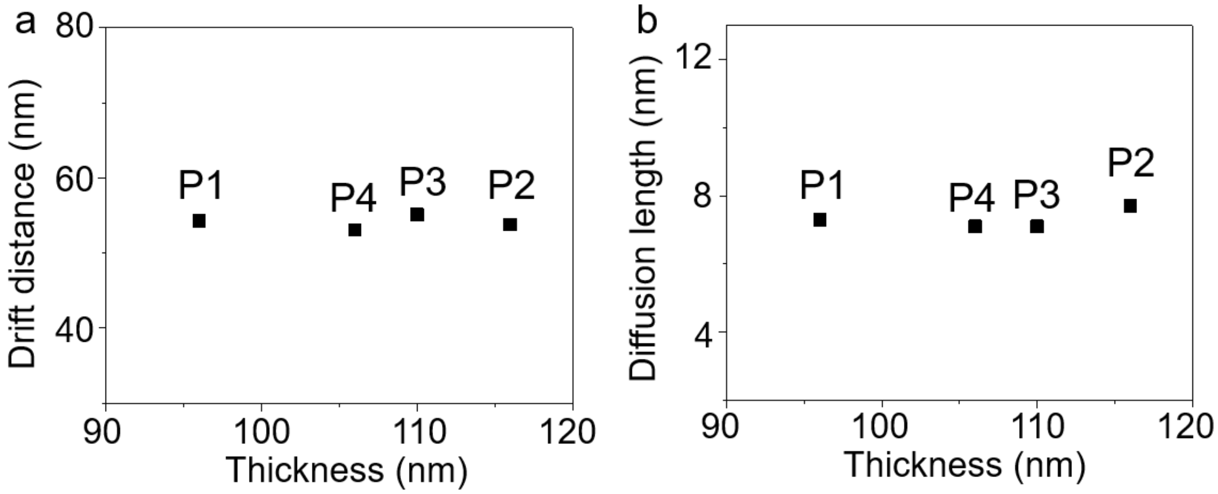

Supplementary Figure 13. The relationship between thickness and charge transfer dynamics for every single BiOBr platelet.
The relationship between thickness and (**a**) drift distance or (**b**) diffusion length for the BiOBr-2.5-P1, P2, P3, and P4 photocatalysts. Source data are provided as a Source Data file.

The relationship between the thickness of BiOBr platelets and its drift distances/diffusion lengths is further plotted in Supplementary Fig. 13. Despite the changes in the thickness of BiOBr-2.5 platelets, the charge dynamics behavior of these BiOBr-2.5 platelets appears in a similar manner (Supplementary Fig. 13), indicating that a change in thickness has little effect on the charge dynamic.

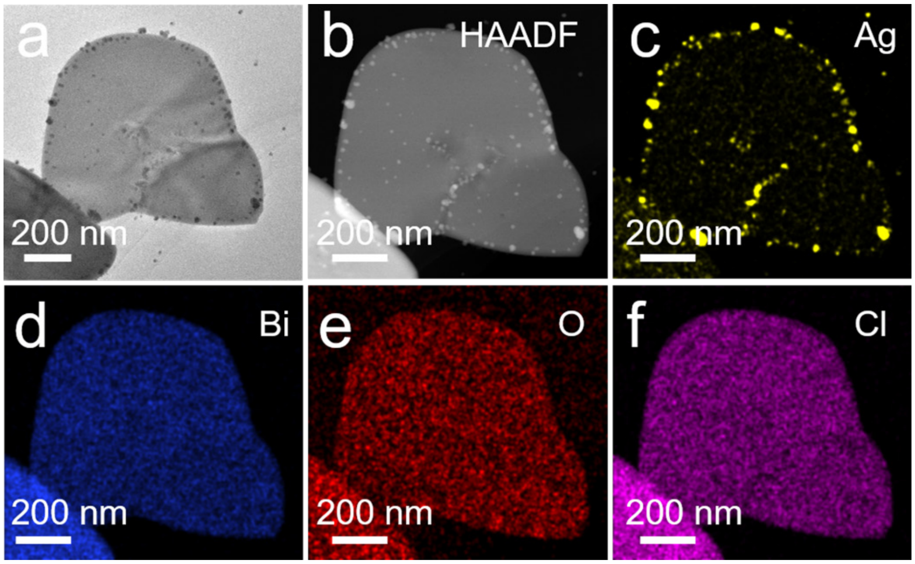

Supplementary Figure 14. Loading Ag nanoparticles on the surfaces of BiOCl platelets by photo-deposition.
(**a**) TEM image, (**b**) HAADF image, and (**c**- **f**) EDS maps of Ag/BiOCl photocatalysts.

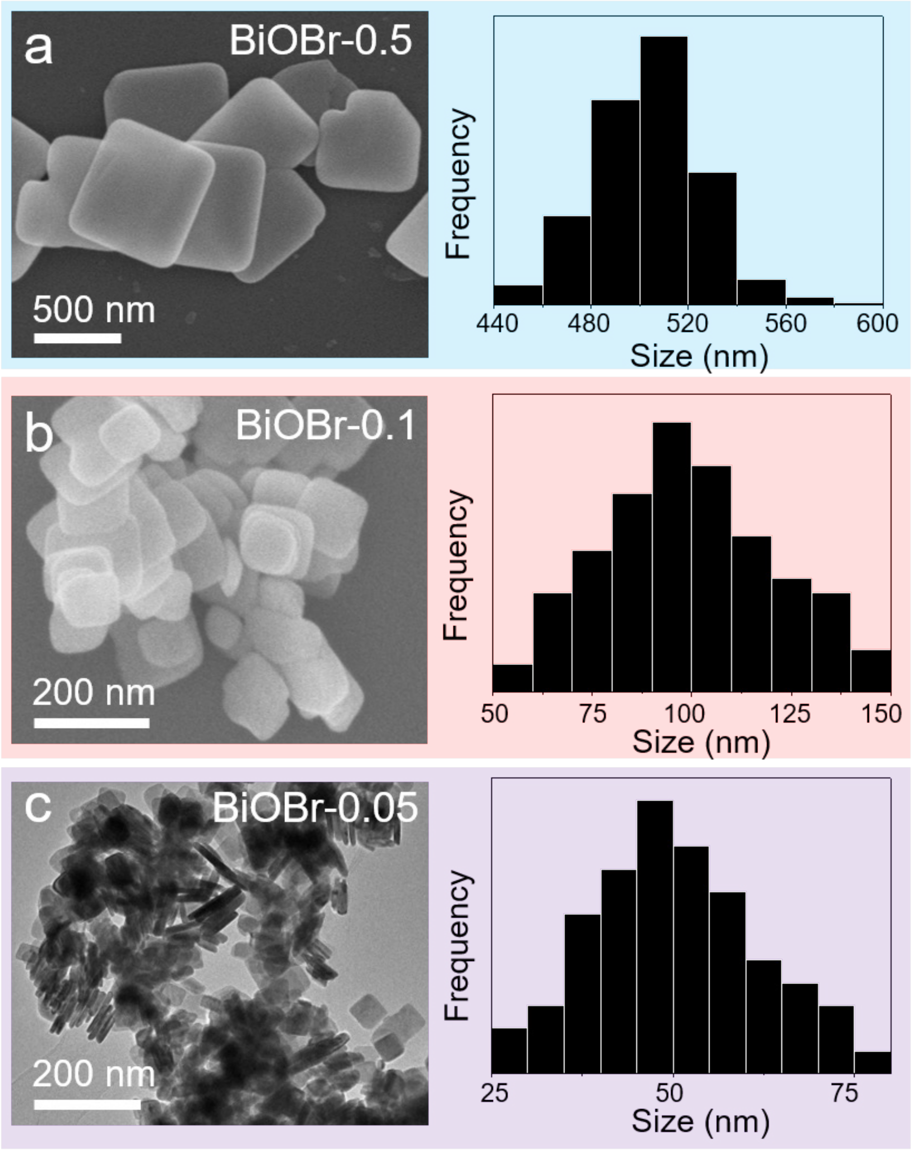

Supplementary Figure 15. BiOBr platelets are produced by different synthetical methods with varied lateral sizes.

SEM, TEM images and statistical histogram of the lateral size of (**a**) BiOBr-0.5, (**b**) BiOBr-0.1, and (**c**) BiOBr-0.05 platelets, respectively. The mean lateral sizes of BiOBr-0.5, BiOBr-0.1, and BiOBr-0.05 are about 500, 100, and 50 nm, respectively. Source data are provided as a Source Data file.

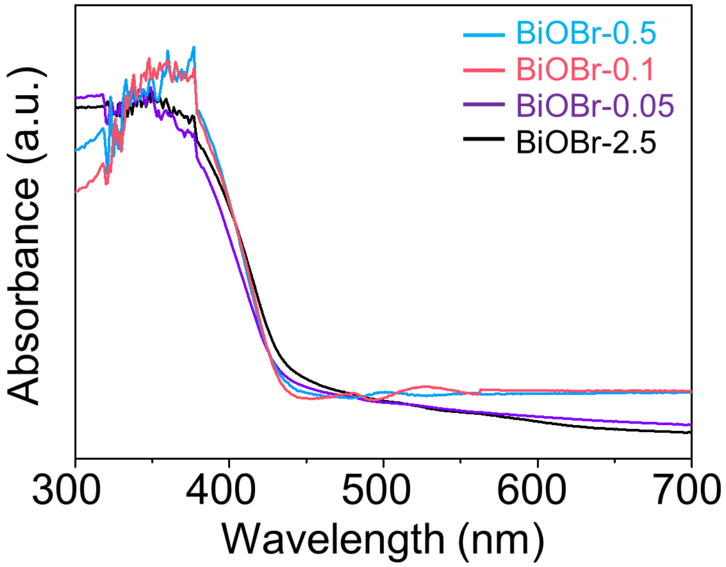

Supplementary Figure 16. DRS spectra of BiOBr-2.5, -0.5, -0.1 and -0.05 photocatalysts. Source data are provided as a Source Data file.

To understand the effect of the optical absorption property of BiOBr platelets on photocatalytic performance, we have added the optical absorption property of BiOBr photocatalysts with different platelet sizes (2500, 500, 100, and 50 nm), which were shown in Supplementary Fig. 16. The results revealed that these BiOBr photocatalysts with different lateral sizes have a similar optical absorption property. Therefore, the photocatalytic activity difference should be caused by variation in charge transport, rather than the light absorption ability.

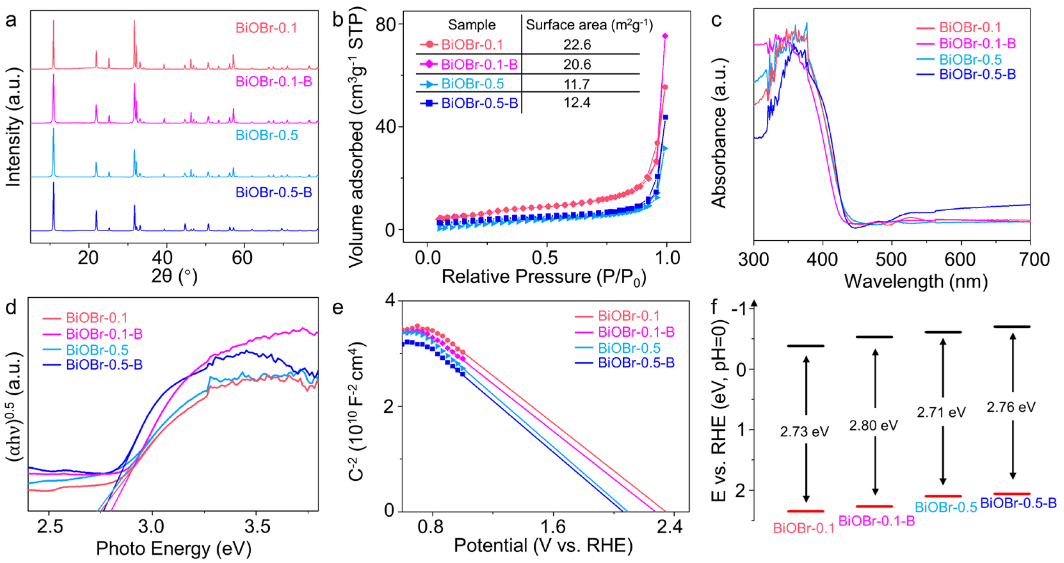

Supplementary Figure 17. Impact of synthetical methods on the chemical, physical and optical properties of BiOBr platelets.
(**a**) XRD patterns, (**b**) BET data, (**c**) DRS spectra, (**d**) Kubelka-Munk transformed reflectance, (**e**) Mott-Schottky plots, and (**f**) band structures of BiOBr-0.1, BiOBr-0.1-B, BiOBr-0.5, and BiOBr-0.5-B photocatalysts. Source data are provided as a Source Data file.

To understand the impact of different synthetical methods on the chemical, physical and optical properties of BiOBr platelets, we have synthesized BiOBr platelets with lateral sizes of 500 and 100 nm by new synthetical methods, and the samples were named BiOBr-0.5-B and BiOBr-0.1-B, respectively (the detailed synthetical method was listed in the Method section). Next, a series of experiments, including the characterizations of XRD, BET, DRS, and Mott-Schottky to estimate the chemical, physical and optical absorption properties of various BiOBr photocatalysts was conducted. These results are presented in Supplementary Fig. 17. The XRD patterns of BiOBr-0.1, BiOBr-0.1-B, BiOBr-0.5, and BiOBr-0.5-B photocatalysts are similar (Supplementary Fig. 17a), indicating that the main structure of various BiOBr photocatalysts using different synthesis methods remains unchanged.

The surface area of various BiOBr photocatalysts is listed in Supplementary Fig. 17b. As expected, BiOBr-0.1 and BiOBr-0.1-B photocatalysts have a larger surface area than BiOBr-0.5 and BiOBr-0.5-B photocatalysts, but this difference is not very significant and is in the same order of magnitude. The optical absorption property, bandgap, and band edge positions of various BiOBr photocatalysts are recorded in Supplementary Fig. 17c-e. As shown in Supplementary Fig. 17c, all of the BiOBr photocatalysts exhibit comparable absorption edges and based on the plots of $(\alpha h\nu)^{0.5}$ versus photon energy (hν) (Supplementary Fig. 17d), the bandgap energies of BiOBr photocatalysts are quite similar, which are calculated to be 2.73, 2.80, 2.71 and 2.76 eV for the BiOBr-0.1, BiOBr-0.1-B, BiOBr-0.5, and BiOBr-0.5-B photocatalysts, respectively. Additionally, the negative slope of the Mott-Schottky plots for BiOBr photocatalysts indicates the p-type characteristics (Supplementary Fig. 17e), and their flat band potentials are measured to be 2.35, 2.27, 2.10, and 2.06 eV for the BiOBr-0.1, BiOBr-0.1-B, BiOBr-0.5, and BiOBr-0.5-B photocatalysts, respectively. Then, the energy band-gap diagram is presented in Supplementary Fig. 17f, implying that all of the BiOBr photocatalysts have appropriate photoredox properties.

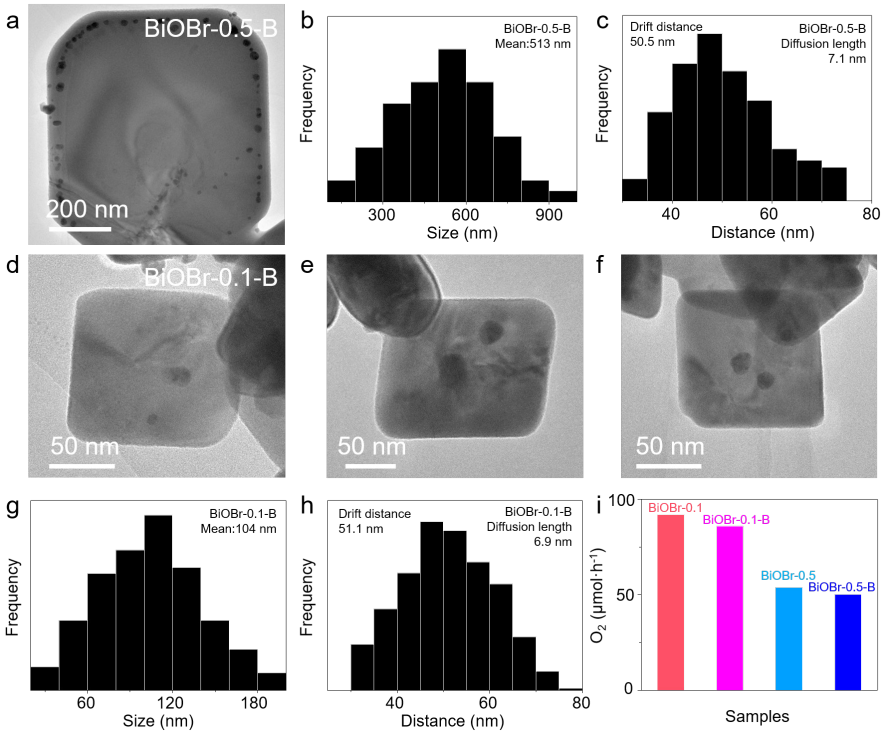

Supplementary Figure 18. Charge transfer dynamics of BiOBr platelets produced by different synthesis methods and their photocatalytic performance. The thickness of BiOBr platelets is fixed to 0.1 and 0.5 μm.
TEM images of photo-deposited Ag nanoparticles for BiOBr platelets produced by different synthesis methods. The corresponding statistical histogram of their lateral sizes and drift distances from the centers of Ag nanoparticles to the edges of BiOBr platelets: (**a-c**) BiOBr-0.5-B, (**d-h**) BiOBr-0.1-B photocatalysts. (**i**) The photocatalytic water oxidation performance of BiOBr-0.1, BiOBr-0.1-B, BiOBr-0.5, and BiOBr-0.5-B photocatalysts. Source data are provided as a Source Data file.

To gain insight into the relationship between different synthesis methods and the behavior of charge dynamics/photocatalytic performance. We also test the charge dynamic and photocatalytic water oxidation performance for these BiOBr photocatalysts with different synthesis methods, and the results are shown in Supplementary Fig. 18. The drift distances of BiOBr-0.5-B and BiOBr-0.1-B are 50.5 and 51.1 nm, respectively. The diffusion lengths of BiOBr-0.5-B and BiOBr-0.1-B are 7.1 and 7.0 nm, respectively. When these results are compared with BiOBr-0.5 (drift distance: 48.7 nm, diffusion length: 7.3 nm) and BiOBr-0.1 (drift distance: 51.8 nm, diffusion length: 7.4 nm) photocatalysts, the differences observed are minor and could be considered negligible, revealing that different synthesis methods have little effect on the charge dynamics of BiOBr photocatalysts. Furthermore, the photocatalytic water oxidation performance of BiOBr photocatalyst is carried out in Supplementary Fig. 18i. The performances of BiOBr-0.1-B and BiOBr-0.5-B photocatalysts are 86 and 50 $\mu mol \cdot h^{-1}$, respectively, which are comparable to the BiOBr-0.1 (93 $\mu mol \cdot h^{-1}$) and BiOBr-0.5 (53.9 $\mu mol \cdot h^{-1}$) photocatalysts, implying that the water oxidation capacities of BiOBr photocatalysts with similar lateral size are close. Therefore, all of these results show that the different synthesis methods have little effect on the chemical, physical, and optical absorption properties of BiOBr photocatalysts as well as the behavior of charge dynamic and their photocatalytic water oxidation performance.

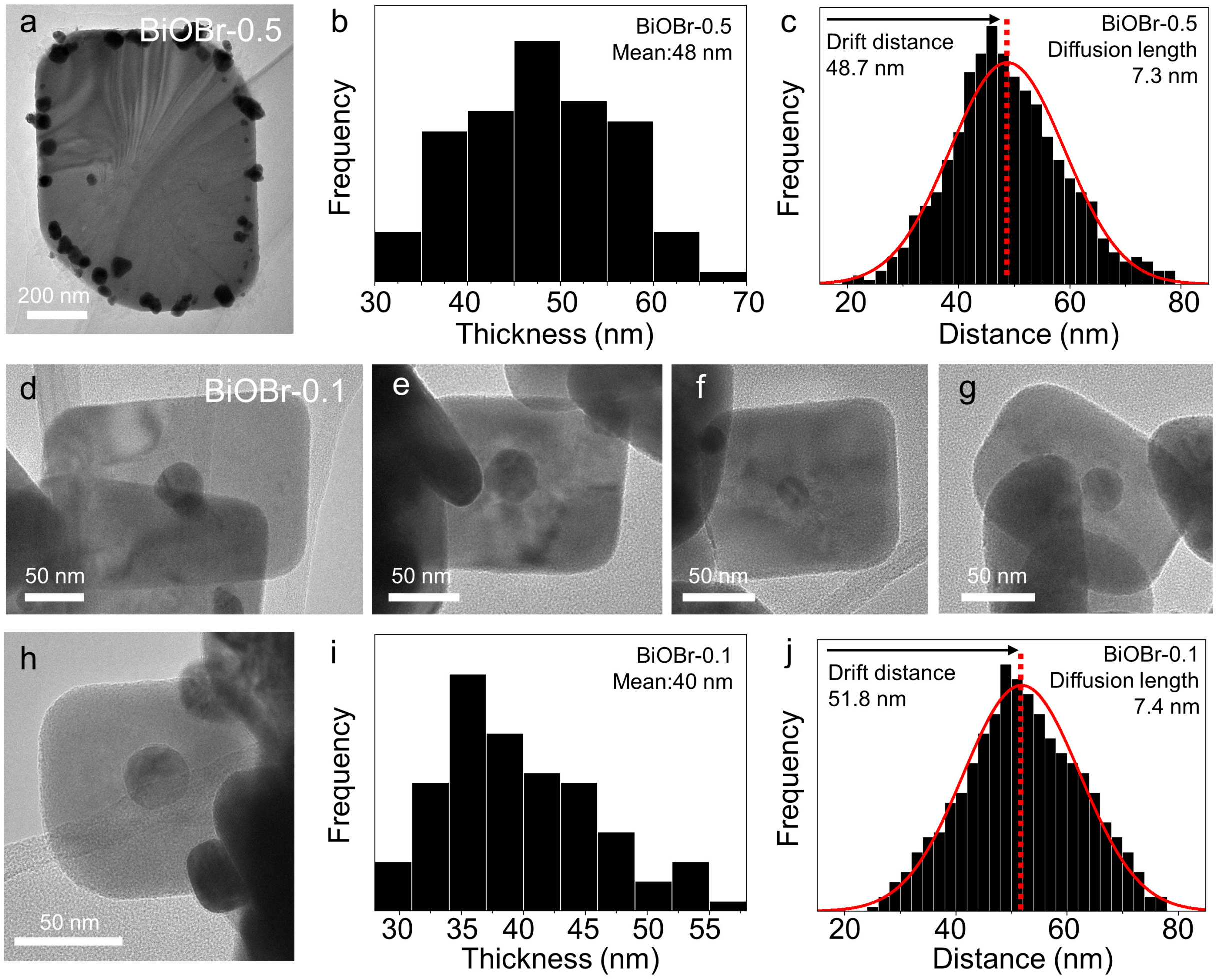

Supplementary Figure 19. The effect of sample thickness on the charge transfer kinetics. TEM images of photo-deposited Ag nanoparticles on the different lateral sizes and thickness of BiOBr platelets with the corresponding statistical histogram of distance from the center of Ag nanoparticles to the edges of BiOBr platelets: (**a**-**c**) BiOBr-0.5, (**d**-**j**) BiOBr-0.1 platelets. Source data are provided as a Source Data file.

To investigate the effect of different lateral sizes on their charge dynamic behaviors, we photo-deposited Ag nanoparticles on the surface of various BiOBr platelets. The thickness, drift distance, and diffusion length of these BiOBr platelets were shown in Supplementary Fig. 19 and Table 1. Lining up of Ag nanoparticles near the platelet edges works for BiOBr-0.5 and BiOBr-0.1 photocatalysts. The drift distance/diffusion length of BiOBr-0.5 and BiOBr-0.1 photocatalysts are 48.7/7.3 (Supplementary Fig. 19a-c) and 51.8/7.4 nm (Supplementary Fig. 19d-j), respectively.

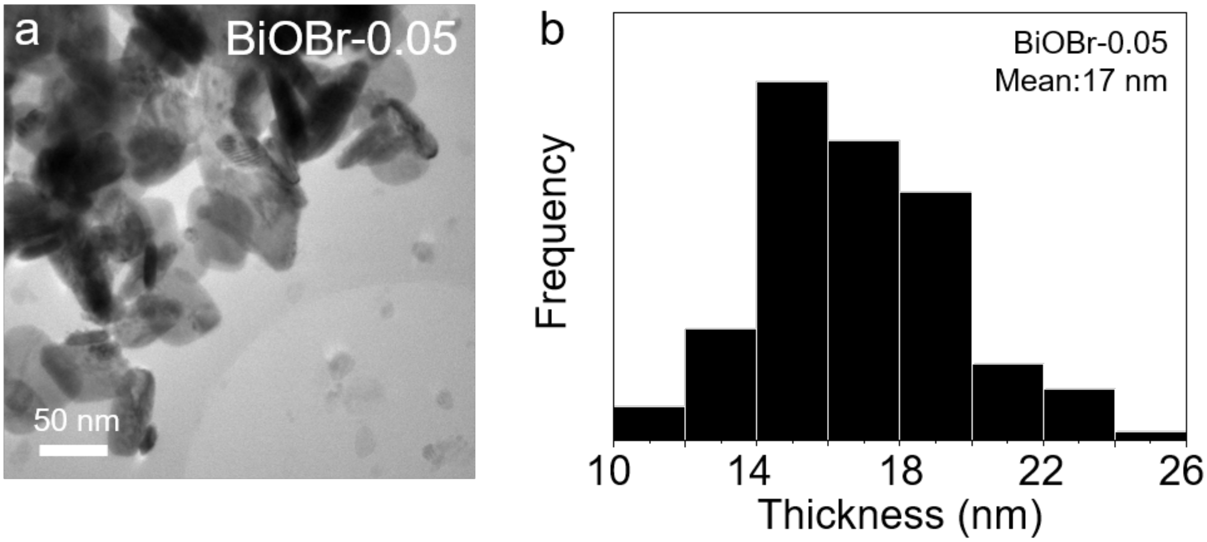

Supplementary Figure 20. Photo-deposition of Ag nanoparticles on the surfaces of BiOBr platelets when their thickness are reduced to ~50 nm.
(**a**) TEM image of photo-deposited Ag nanoparticles on the BiOBr-0.05 platelets with the corresponding statistical histogram of its (**b**) thickness. Note that Ag nanoparticles with dark contrasts randomly distribute on the surface of BiOBr-0.05 platelets and the carbon film of the Cu grid, this might be due to a reduction of platelet thickness (mean thickness: 17 nm), which decreases the width of the space charge region in the thickness direction, weakening the strength of IEF_z [12]. The internal electric field within the facet junction became weaker to drive the directional migration of charge carriers, leading to a random distribution of Ag nanoparticles as probes of photo-generated electrons. Source data are provided as a Source Data file.

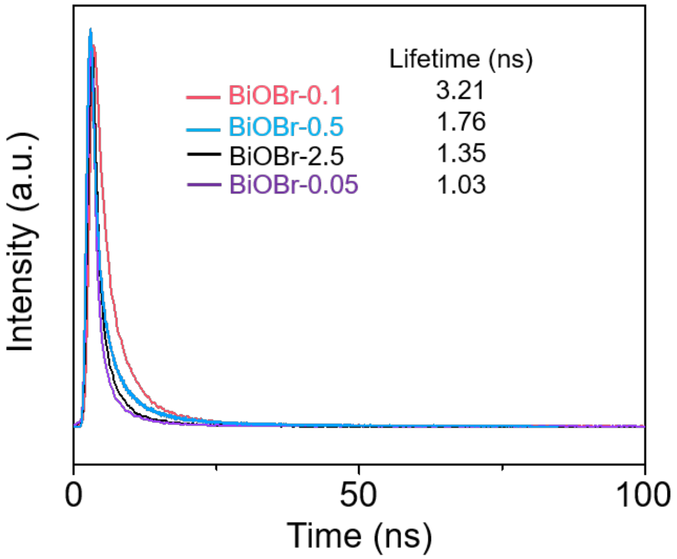

Supplementary Figure 21. Time-resolved lifetime measurements of BiOBr-2.5, -0.5, -0.1 and -0.05 photocatalysts.
Source data are provided as a Source Data file.

To estimate the charge mobility in BiOBr systems, we tested the time-resolved lifetime measurements of BiOBr platelets (Supplementary Fig. 21). The overall lifetime of electrons for BiOBr-0.05, 0.1, 0.5 and 2.5 photocatalysts are 1.03, 3.21, 1.76, and 1.03 ns, respectively.

Taking the BiOBr-2.5 photocatalyst as an example, the value of its overall electron lifetime is 1.35 ns. Thus, the actual flight time of electrons (τ_1) with the facet junction should be less than 1.35 ns. Here we assume that τ_1 is close to 1.35 ns.

For the value of electric field (ε_x), we assume that it is a uniform electric field. The strength of an electric field is $\Delta V/d$, where ΔV is the potential difference and d is the distance. ΔV is related to the surface voltage[13], which is 0.1mV from the previous literatures[14, 15]. For the value of d, we assume that d is equivalent to the drift distance of electrons (~55.7 nm).

Finally, using the equation of $\mu = L_{IEF_x}/(\varepsilon_x \tau_1) = (L_{IEF_x} \times d)/(\Delta V \times \tau_1)$, and the μ is derived as $2.30 \times 10^2\ cm^2/(V \cdot s)$. The theoretical charge mobility is $5.33 \times 10^2\ cm^2/(V \cdot s)$[16], which is basically in the same magnitude compared to our results, revealing that our estimation is reliable.

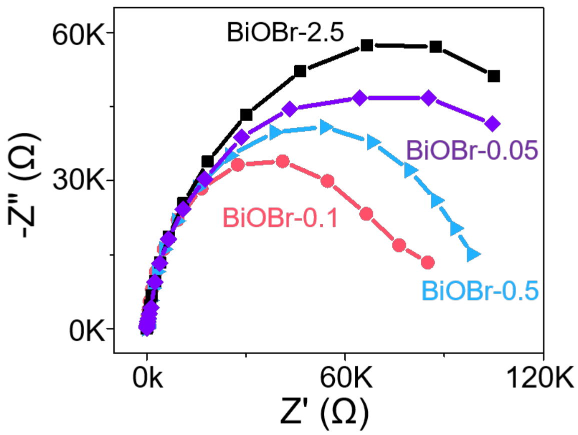

Supplementary Figure 22. Electrochemical impedance spectroscopy (EIS) spectra of BiOBr-2.5, -0.5, -0.1 and -0.05 platelets.
Source data are provided as a Source Data file.

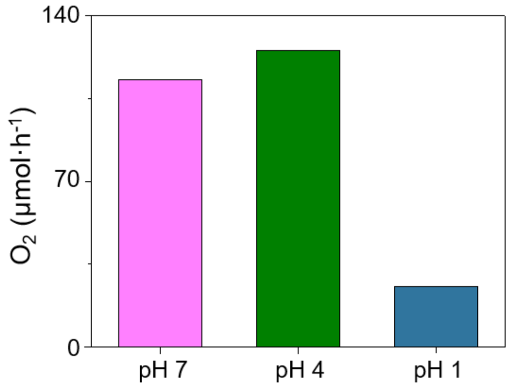

Supplementary Figure 23. Photocatalytic performance of BiOBr-0.1 platelets under different pH values conditions.

The pH-dependent water oxidation evolution performance of Pt-BiOBr-0.1 platelets, using $Fe(NO_3)_3 \cdot 9H_2O$ as an electron acceptor ($Fe^{3+} + e^- \rightarrow Fe^{2+}$) and the dilute nitric acid as a pH regulator. The lateral size of Pt-BiOBr-0.1 platelets is ca. 100 nm. Using Pt as electron probes, the mean drift distances of electrons at pH=7, 4, and 1 are 33.6, 41.5, and 52.1 nm, respectively (Supplementary Table 1). It is found that the performance at pH=7 is lower than that at pH=4, where the lateral grain size is closer to twice the drift distance (100 nm versus 2*41.5 nm). Notably, when the pH value is set at 1, the mean lateral size (~100 nm) is almost twice the drift distance (104.2 nm). Unfortunately, at this pH value, a very low water oxidation performance is exhibited. This discrepancy is because, as the pH value decreases, the oxidability of HNO_3 increases, which prohibits $Fe(NO_3)_3 \cdot 9H_2O$ from capturing electrons. Source data are provided as a Source Data file.

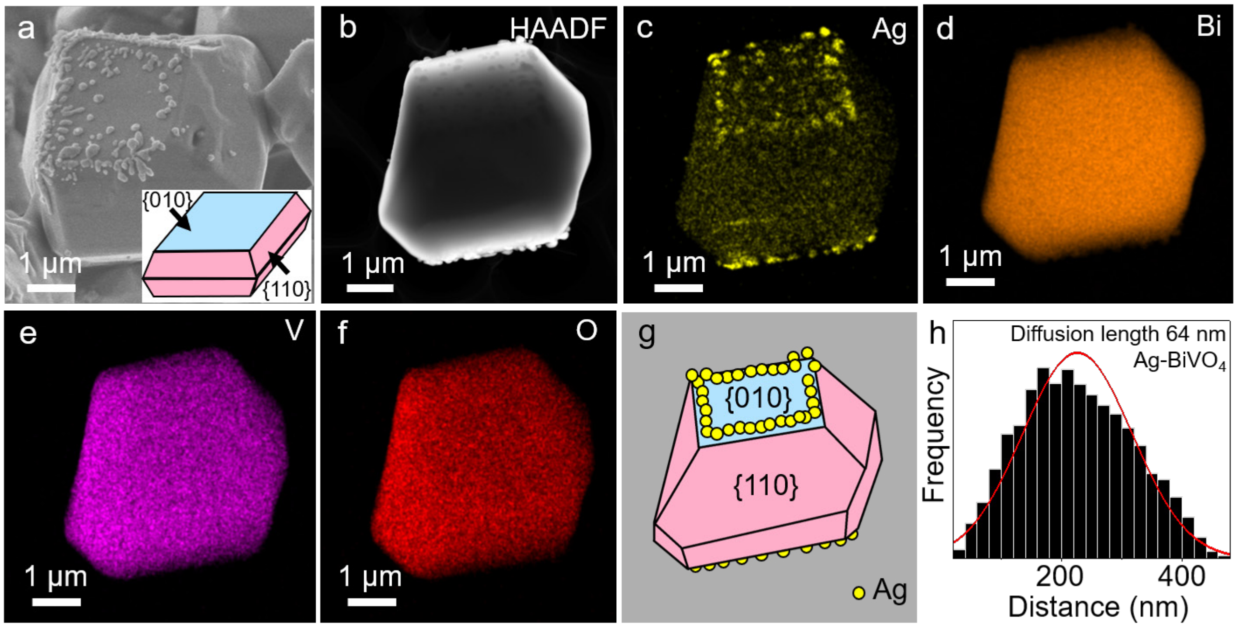

Supplementary Figure 24. Collective charge transfer migration steered by IEFs for $BiVO_4$ photocatalysts.

(**a**) SEM, (**b-f**) EDS maps of photo-deposited $BiVO_4$ photocatalysts. Note that Ag nanoparticles have a propensity to accumulate around the edges of reductive {010} facets for $BiVO_4$ photocatalysts, forming distinct lines, as clearly shown in a schematic diagram of (**g**). (**h**) The corresponding statistical histogram of distances from the centers of Ag nanoparticles to the edges of $BiVO_4$ photocatalysts, showing a characteristic Gaussian curve distribution. The diffusion length of electrons within $BiVO_4$ photocatalysts is estimated to be 64 nm. Source data are provided as a Source Data file.

To examine the charge dynamic behavior in $BiVO_4$ photocatalysts, we also photo-deposited Ag nanoparticles on their surfaces. As shown in Supplementary Fig. 24, Ag nanoparticles accumulate around the edges of the top facets of {010} for $BiVO_4$ photocatalysts (Supplementary Fig. 24a-g). After statistical analysis of the distribution for Ag nanoparticles in the $Ag/BiVO_4$ system, the diffusion length of electrons within $Ag/BiVO_4$ photocatalysts is 64 nm (Supplementary Fig. 24h), which is close to the value of ~70 nm reported in the literature[17].

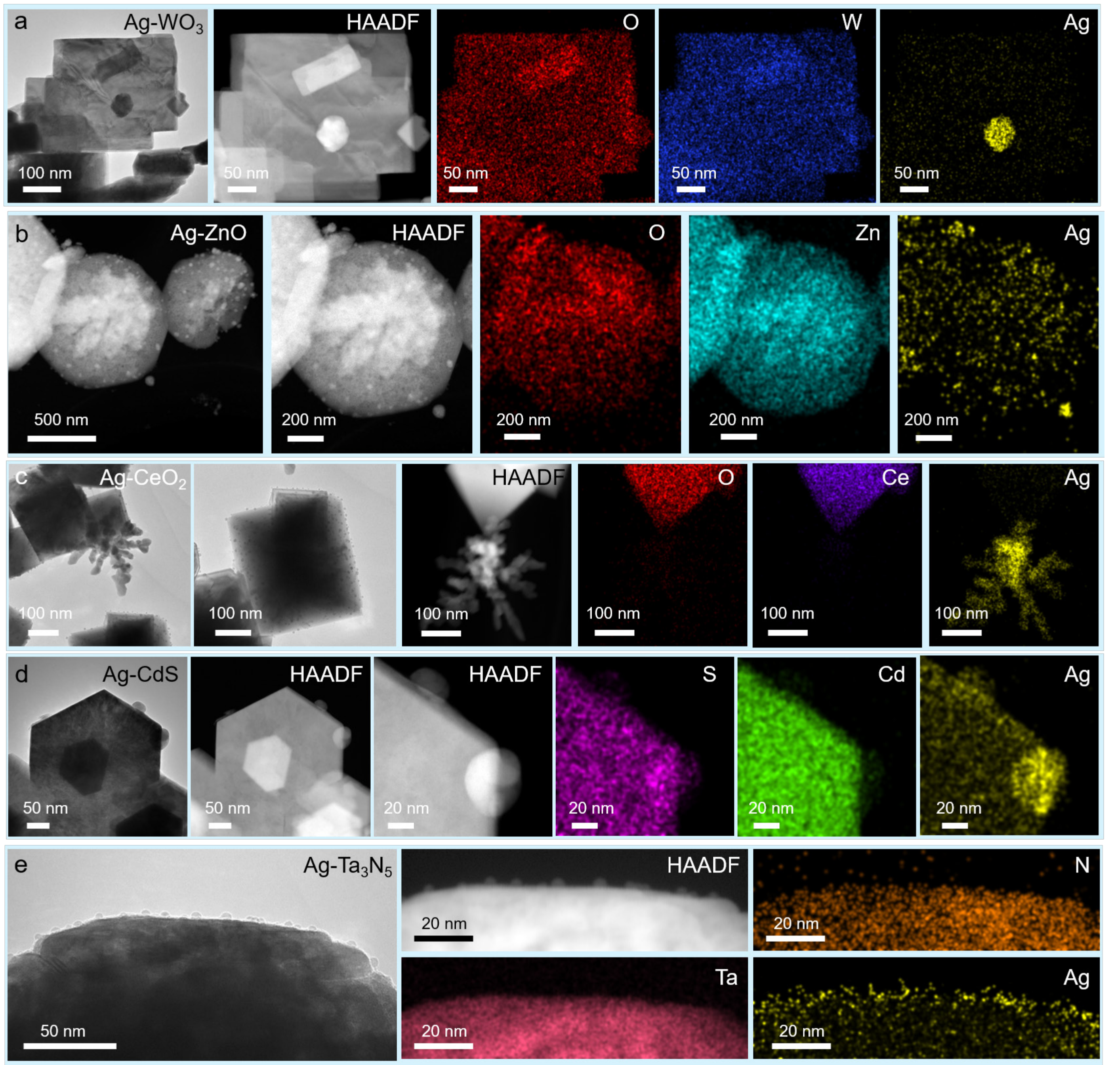

Supplementary Figure 25. Photo-deposition of Ag nanoparticles on the surfaces of some common photocatalysts.
TEM images and EDS maps of photo-deposited Ag nanoparticles on (**a**) WO_3, (**b**) ZnO, (**c**) CeO_2, (**d**) CdS, and (**e**) Ta_3N_5 photocatalysts. Note that Ag nanoparticles randomly distributed on the surface of these photocatalysts.

Ag nanoparticles were also photo-deposited on the surface of WO_3, ZnO, CeO_2, CdS, and Ta_3N_5 photocatalysts. As shown in Supplementary Fig. 25, Ag nanoparticles randomly dispersed on the surface of WO_3, ZnO, CeO_2, CdS, and Ta_3N_5 photocatalysts without forming a regular clustering pattern. To understand the observed photo-deposition differences, we have investigated the literature and found that the $BiVO_4$ photocatalysts have two spatially separated reductive {010} and oxidative {110} surfaces[18]. The formation of {010}/{110} facet junctions (defined as a homojunction between neighboring facets in a single-crystalline semiconductor)[3-5] is beneficial for expediting charge separation. Facet junctions were not observed for the above photocatalysts. Hence, we propose this approach might be applicable to photocatalysts with spatially separated reductive and oxidative surfaces.

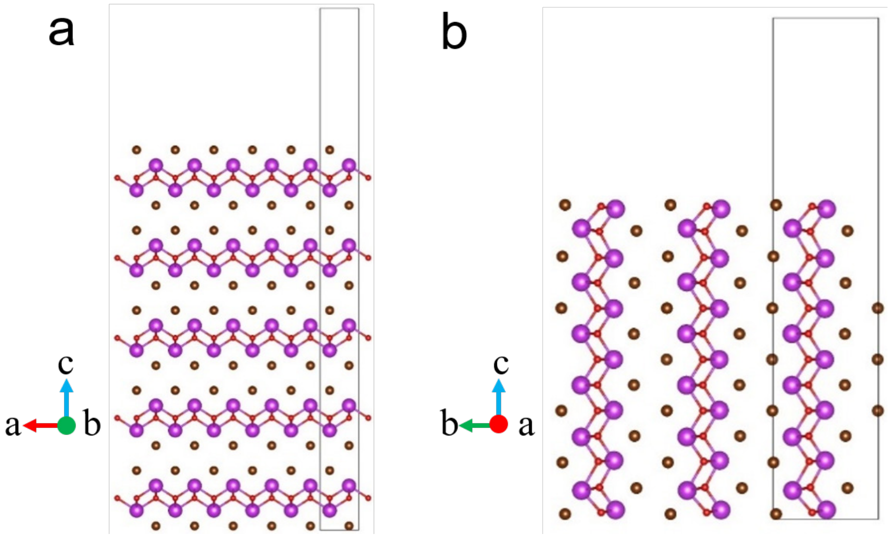

Supplementary Figure 26. Atomic models of (001) and (200) surfaces of BiOBr photocatalysts. Atomic models containing (**a**) (001) and (**b**) (200) surfaces for bandgap calculation. The black boxes show the primitive unit cells with vacuum slabs for DFT calculation. The **a**, **b**, and **c** arrows indicate [100], [010] and [001] directions. Red, brown, and purple spheres represent O, Br, and Bi adatoms.

Supplementary Table

Supplementary Table 1. A summary of the lateral drift distances and the diffusion lengths of electrons using MnO_x nanoparticles or solutions with varying pH values to construct lateral IEFs. Source data are provided as a Source Data file.

Samples	Lateral drift distance (L_{IEFx}, nm)	Standard deviation (σ, nm)	Diffusion length (L_e, nm)
MnO_x-Ag/BiOBr	51.9	10.5	7.4
MnO_x-Pt/BiOBr	47.5	9.9	7.0
MnO_x-Au/BiOBr	33.0	10.1	7.1
Pt(pH=7)/BiOBr	33.6	9.4	6.6
Pt(pH=4)/BiOBr	41.5	9.9	7.0
Pt(pH=1)/BiOBr	52.1	10.6	7.5

Supplementary References

1. Qu, Z. *et al.* Study of the structure, electronic and optical properties of BiOI/Rutile-TiO_2 heterojunction by the first-principle calculation. *Materials* **13**, (2020).
2. Wang, J. *et al.* Optoelectronic response and interfacial properties of BiOI/BiOX (X=F, Cl, Br) heterostructures based on DFT investigation. *J. Solid State Chem.* **284**, 121181 (2020).
3. Sun, S. *et al.* Facet junction engineering for photocatalysis: A comprehensive review on elementary knowledge, facet-synergistic mechanisms, functional modifications, and future perspectives. *Adv. Funct. Mater.* **32**, 2106982 (2022).
4. Yu, J. *et al.* Enhanced photocatalytic CO_2-reduction activity of anatase TiO_2 by coexposed {001} and {101} facets. *J. Am. Chem. Soc.* **136**, 8839-8842 (2014).
5. Zhang, A.-Y. *et al.* Epitaxial facet junctions on TiO_2 single crystals for efficient photocatalytic water splitting. *Energy Environ. Sci.* **11**, 1444-1448 (2018).
6. Parrott, J. E. The theory of majority-carrier motion in the Haynes-Shockley experiment. *Solid-State Electron.* **28**, 1065-1075 (1985).
7. Shacham‐Diamand, Y. & Kidron, I. Haynes-Shockley experiment on n-type HgCdTe. *J. Appl. Phys.* **56**, 1104-1108 (1984).
8. Egerton, R. F. *Electron Energy-Loss Spectroscopy in the Electron Microscope*. Springer, 2011.
9. Malis, T., Cheng, S. C. & Egerton, R. F. EELS log-ratio technique for specimen-thickness measurement in the TEM. *J. Electron Microsc. Tech.* **8**, 193-200 (1988).
10. Lee, C.-W., Ikematsu, Y. & Shindo, D. Thickness measurement of amorphous SiO_2 by EELS and electron holography. *Mater. Trans., JIM* **41**, 1129-1131 (2000).
11. Heo, Y.-U. Comparative study on the specimen thickness measurement using EELS and CBED methods. *Appl. Microsc.* **50**, 8 (2020).
12. Rothenberger, G. *et al.* Charge carrier trapping and recombination dynamics in small semiconductor particles. *J. Am. Chem. Soc.* **107**, 8054-8059 (1985).
13. Li, J. *et al.* Giant enhancement of internal electric field boosting bulk charge separation for photocatalysis. *Adv. Mater.* **28**, 4059-4064 (2016).
14. Jia, H. *et al.* Mechanistic insights into the photoinduced charge carrier dynamics of BiOBr/CdS nanosheet heterojunctions for photovoltaic application. *Nanoscale* **9**, 3180-3187 (2017).
15. Jia, H. *et al.* Facile synthesis of bismuth oxyhalide nanosheet films with distinct conduction type and photo-induced charge carrier behavior. *Appl. Surf. Sci.* **441**, 832-840 (2018).
16. Yu, J., Li, T. & Sun, Q. Single-layer BiOBr: An effective p-type 2D thermoelectric material. *J. Appl. Phys.* **125**, 205111 (2019).
17. Abdi, F. F. *et al.* The origin of slow carrier transport in $BiVO_4$ thin film photoanodes: A time-resolved microwave conductivity study. *J. Phys. Chem. Lett.* **4**, 2752-2757 (2013).
18. Li, R. *et al.* Spatial separation of photogenerated electrons and holes among {010} and {110} crystal facets of $BiVO_4$. *Nat. Commun.* **4**, 1432 (2013).
